# Spatial and Temporal Dynamics of Prokaryotic and Viral Community Assemblages in a Lotic System (Manatee Springs, Florida)

**DOI:** 10.1128/AEM.00646-21

**Published:** 2021-08-26

**Authors:** Kema Malki, Natalie A. Sawaya, Michael J. Tisza, Felipe H. Coutinho, Karyna Rosario, Anna J. Székely, Mya Breitbart

**Affiliations:** a College of Marine Science, University of South Florida, Saint Petersburg, Florida, USA; b Laboratory of Cellular Oncology, NCI, NIH, Bethesda, Maryland, USA; c Departamento de Produccíon Vegetal y Microbiología, Universidad Miguel Hernández, San Juan de Alicante, Spain; d Department of Ecology and Genetics/Limnology, Uppsala University, Uppsala, Sweden; e Department of Aquatic Sciences and Assessment, Swedish University of Agricultural Sciences, Uppsala, Sweden; Norwegian University of Life Sciences

**Keywords:** freshwater, environmental microbiology, virome, springs, prokaryote, virus, microbial ecology, lotic systems, community coalescence

## Abstract

Flow from high-magnitude springs fed by the Floridan aquifer system contributes hundreds of liters of water per second to rivers, creating unique lotic systems. Despite their importance as freshwater sources and their contributions to the state’s major rivers, little is known about the composition and spatiotemporal variability of prokaryotic and viral communities of these spring systems or their influence on downstream river sites. At four time points throughout a year, we determined the abundance and diversity of prokaryotic and viral communities at three sites within the first-magnitude Manatee Springs system (the spring head where water emerges from the aquifer, a mixed region where the spring run ends, and a downstream site in the Suwannee River). The abundance of prokaryotes and virus-like particles increased 100-fold from the spring head to the river and few members from the head communities persisted in the river at low abundance, suggesting the springs play a minor role in seeding downstream communities. Prokaryotic and viral communities within Manatee Springs clustered by site, with seasonal variability likely driven by flow. As water flowed through the system, microbial community composition was affected by changes in physiochemical parameters and community coalescence. Evidence of species sorting and mass effects could be seen in the assemblages. Greater temporal fluctuations were observed in prokaryotic and viral community composition with increasing distance from the spring outflow, reflecting the relative stability of the groundwater environment, and comparisons to springs from prior work reaffirmed that distinct first-magnitude springs support unique communities.

**IMPORTANCE** Prokaryotic and viral communities are central to food webs and biogeochemical processes in aquatic environments, where they help maintain ecosystem health. The Floridan aquifer system (FAS), which is the primary drinking water source for millions of people in the southeastern United States, contributes large amounts of freshwater to major river systems in Florida through its springs. However, there is a paucity of information regarding the spatiotemporal dynamics of microbial communities in these essential flowing freshwater systems. This work explored the prokaryotic and viral communities in a first-magnitude spring system fed by the FAS that discharges millions of liters of water per day into the Suwannee River. This study examined microbial community composition through space and time as well as the environmental parameters and metacommunity assembly mechanisms that shape these communities, providing a foundational understanding for monitoring future changes.

## INTRODUCTION

Diminishment of freshwater resources is a growing issue worldwide. An expanding global population and the development associated with population growth have drastically increased the demand for freshwater in the last century (1). Rivers and groundwater are major sources of freshwater, with groundwater sources such as aquifers representing the second largest reservoir of freshwater on Earth (2). Prokaryotic and viral populations in aquatic ecosystems contribute to nutrient cycling and aid in maintaining ecosystem health through the removal of excess nutrients and pollutants. Compared to other aquatic environments, there is a paucity of research on the microbiology of lotic systems (i.e., rapidly moving freshwater systems) (3). The available research has established hydrology, among other factors, as important in shaping these communities, with flow rates impacting the residence time, introduction, and dispersal of community members and nutrients (4–6).

The Floridan aquifer is one of the most productive aquifers in the world and provides potable water to roughly 10 million people (7). The Floridan aquifer system (FAS) is intimately connected to major river systems throughout the state of Florida. Through high-magnitude springs, flow from the aquifer contributes hundreds of millions of liters of freshwater to river systems each day, creating unique lotic systems (8). The large groundwater input from the springs plays a major role in influencing downstream conditions in river systems, impacting nutrient concentrations and potentially introducing novel groundwater prokaryotes and viruses to the rivers. Although there are studies investigating nutrient loading in aquifers and downstream rivers (9, 10), little is known about the influence of the spring’s prokaryotic and viral communities on a river’s microbiome and virome. Do introduced microbial and viral inocula persist? If so, what proportion of taxa present in rivers are seeded from the springs? Since microbial inhabitants form the foundation of aquatic ecosystems and are essential for maintaining ecosystem health, it is important to understand microbial community dynamics within these systems (11–14).

Our previous work examined the prokaryotic and viral communities of the FAS, generating a snapshot of five first-magnitude springs at the point of discharge from the aquifer (15). The prokaryotic and viral communities in each of the springs were unique, likely due to the influence of differing land usage in their springsheds. However, the changes that occur in these communities as water flows downriver and their stability over time remain unknown. Baseline information regarding the spatiotemporal dynamics of prokaryotic and viral communities in river systems fed by springs is needed to understand the mechanisms that play a role in shaping microbial metacommunities in these understudied lotic systems.

This study investigated prokaryotic and viral community dynamics in the first-magnitude Manatee Springs system in northwestern Florida. Discharging ∼378 million liters of water each day, the flow from this spring is a major tributary to the southern Suwannee River (8). Four times over the course of a year, we sampled three sites within the system: the spring discharge (head), the site where the spring run meets the river (mixed), and a downstream point in the Suwannee River (river). We hypothesized that location within the system would play a greater role than sampling date in driving prokaryotic and viral community composition and that greater temporal fluctuations would be observed with increasing distance from the spring outflow due to the stability of the groundwater environment. To test these hypotheses, we measured basic physiochemical parameters, determined the abundance of prokaryotic cells and virus-like particles (VLPs) by epifluorescence microscopy, and sequenced 16S rRNA gene amplicons and viral metagenomes (viromes) from each sample. This study contributes to our knowledge of prokaryotic and viral ecology in lotic systems, specifically deepening our understanding of rivers and vital spring systems of the state of Florida and the southeastern United States.

## RESULTS

### Physiochemical parameters.

**FIG 1:**
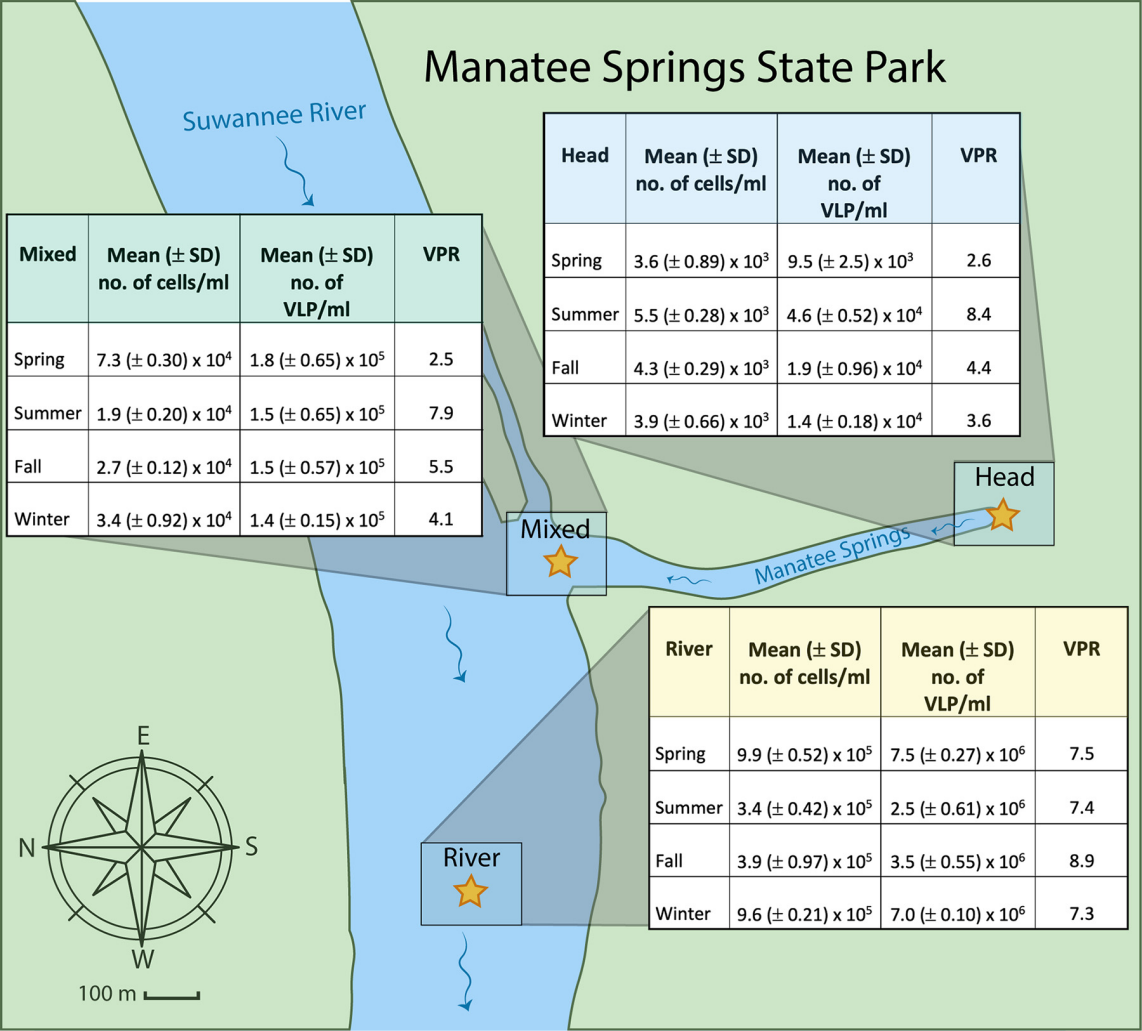
Map of Manatee Springs State Park depicting the three sites (head, mixed, and river). Tables contain concentrations of prokaryotic cells and virus-like particles (VLP) determined through epifluorescence microscopy along with the calculated virus-to-prokaryote ratios (VPR).

Three sites (the spring head, the mixed region, and a downstream river site) with distinct hydrogeological features within the Manatee Springs system were sampled four times (spring, summer, fall, and winter) between May 2017 and January 2018 (Fig. 1; see also Table S1 in the supplemental material). Temperature at the spring head was consistent over time. Temperature and pH in the mixed and river samples varied temporally, although both parameters varied more between the sampling sites than across time points. Dissolved oxygen (DO) concentrations were lowest in the samples collected from the spring head (14.0 to 30.1%) and highest in the river samples (88.7 to 98.2%) at all time points. In most cases, phosphate, ammonium, and nitrite concentrations increased with distance from the spring head. Conductivity measurements were highest in the head samples (504 to 547 μS/cm) and lowest in the river samples (312 to 378 μS/cm). For all time points except summer, the conductivity of the head and mixed site were almost identical, while in the summer the conductivity of the mixed region deviated slightly toward the river’s conductivity, suggesting higher mixing between these two sites.

### System hydrology and precipitation.

Hydrology and precipitation data were acquired from the United States Geological Survey. Upstream river discharge rates were 4,360 ft^3^/s during the spring, 7,450 ft^3^/s during the summer, 7,000 ft^3^/s during the fall, and 4,100 ft^3^/s during the winter. Likewise, spring discharge rates were 142.5 ft^3^/s during the spring, 127.0 ft^3^/s during the summer, 186.5 ft^3^/s during the fall, and 147.5 ft^3^/s during the winter (Table S1). Cumulative precipitation totals were calculated for the week before the sampling dates and were 0.48 in. in spring, 2.53 in. in summer, 0.26 in. in fall, and 0.01 in. in winter. The highest river flow and lowest spring flow rates are both seen in the summer, corresponding to the increase in precipitation during the rainy season.

### Abundance of prokaryotic cells and VLPs.

SYBR Gold staining and epifluorescence microscopy demonstrated increasing concentrations of prokaryotic cells and VLPs with distance from the spring outflow, with the mixed region containing approximately an order of magnitude more than the spring head, and concentrations at the river site were 2 orders of magnitude greater than those at the spring head. At the head of the springs, values ranged from 3.6 × 10^3^ to 5.5 × 10^3^ cells/ml to 9.5 × 10^3^ to 1.4 × 10^4^ VLP/ml, mixed region values ranged from 1.9 × 10^4^ to 7.3 × 10^4^ cells/ml to 1.4 × 10^5^ to 1.8 × 10^5^ VLP/ml, and the river site contained 3.4 × 10^5^ to 9.9 × 10^5^ cells/ml and 2.5 × 10^6^ to 7.5 × 10^6^ VLP/ml (Fig. 1). While these abundances varied with the date of collection, no clear seasonal trends were observed.

### Prokaryotic community composition.

**FIG 2:**
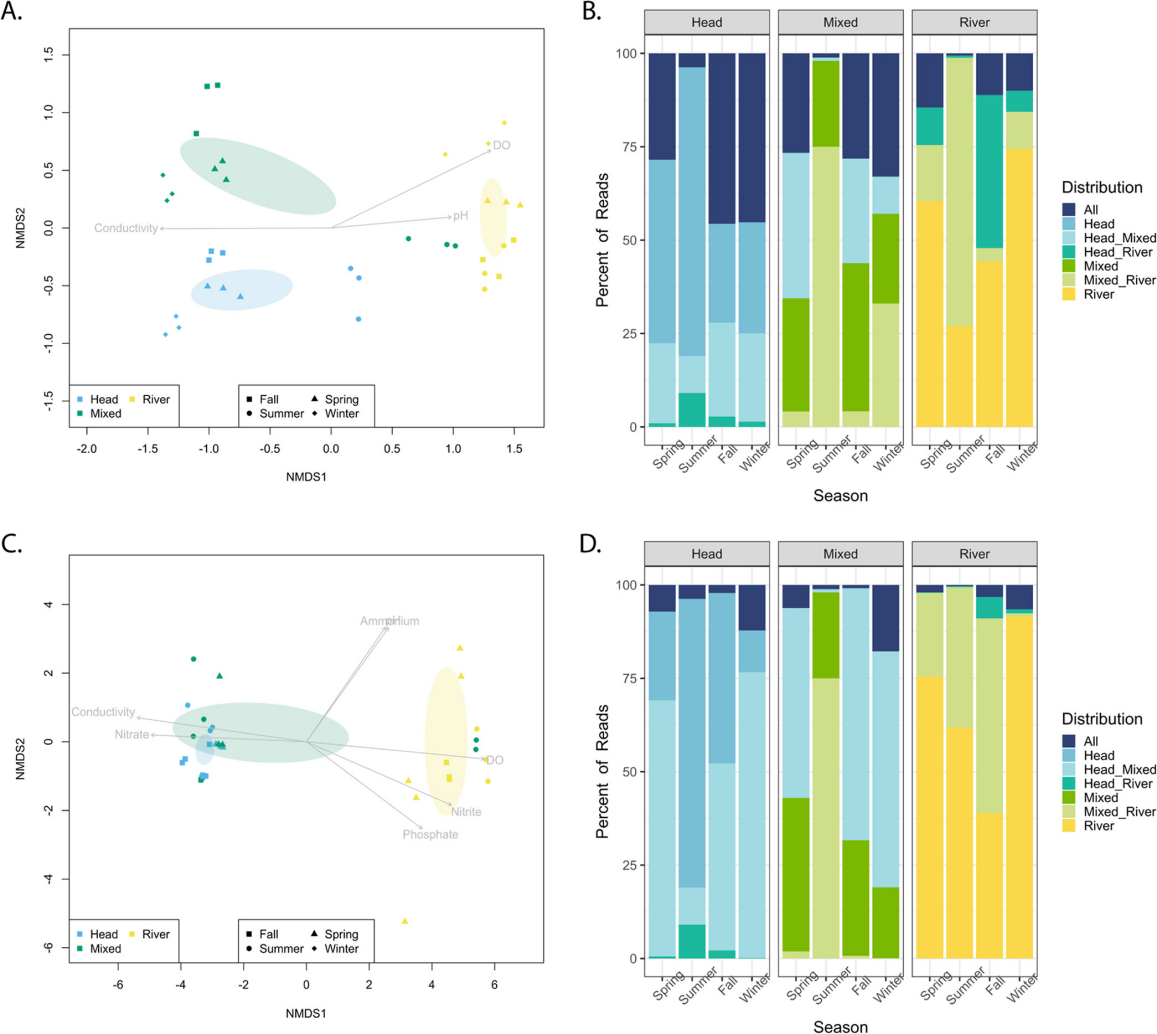
NMDS plots showing the similarity of the prokaryotic (A) and viral (C) community structure of each site through time based on a Bray-Curtis dissimilarity matrix of the relative abundance of ASVs and viral contigs, respectively. In both plots, sampling season and site are denoted by shape and color, respectively, and ordinal ellipses represent the 95% confidence interval of the standard error. The arrows depict the statistically significant physiochemical parameters (*P* < 0.01), and the length represents the magnitude of influence on the ordination of the communities. Stacked barplots show the percentage of mappable reads in each sample belonging to ASVs (B) and viral contigs (D) found in all sites, in a single site, and shared between two sites.

Amplicon sequencing of 16S rRNA genes from the Manatee Springs samples resulted in 5,673 amplicon sequence variants (ASVs). A nonmetric multidimensional scaling (NMDS) plot based on the Bray-Curtis dissimilarity of the relative abundances of ASVs in each sample, with a stress value of 0.12, generally showed grouping by site (Fig. 2A). The ordinal ellipses, representing the 95% confidence interval of the standard error, did not overlap, indicating distinct prokaryotic community means for each site. In contrast to the other seasons, the summer samples from the head and mixed regions showed increased similarity to the river samples. These temporal patterns were consistent with the physiochemical parameters that indicated more mixing between these sites in summer (Table S1). The statistically significant physiochemical parameters (conductivity, DO, and pH) were mapped onto the NMDS. A two-way permutational analysis of variance (PERMANOVA) was conducted to account for both season and site; both were statistically significant (*P* = 0.001, *R*^2^ = 0.27 and *P* = 0.001, *R*^2^ = 0.31, respectively). The interaction between the two variables was also significant (*P* = 0.001, *R*^2^ = 0.25), indicating that both season and distance from the spring outflow played a role in shaping community structure with an additional interactive effect.

The majority of ASVs were unique to a single site (89%) and season (78%). Very few ASVs were shared among all three sites, with only approximately 2% of ASVs found in all sites in the spring, fall, and winter samples; however, this percentage increased in the summer, where 9% of ASVs were shared among all sites (Fig. S1A). In the spring, fall, and winter samples, the head and mixed sites had the largest number of shared ASVs, comprising between 5.5 and 8.0% of the total number of ASVs for each season. In contrast, in the summer, the river and mixed sites shared more ASVs than the head and mixed sites, and more ASVs were shared between all sites than any two sites. Further demonstrating the temporal variation in prokaryotic communities, less than 4% of ASVs from a given site were shared across all seasons (Fig. S2A).

**FIG 3:**
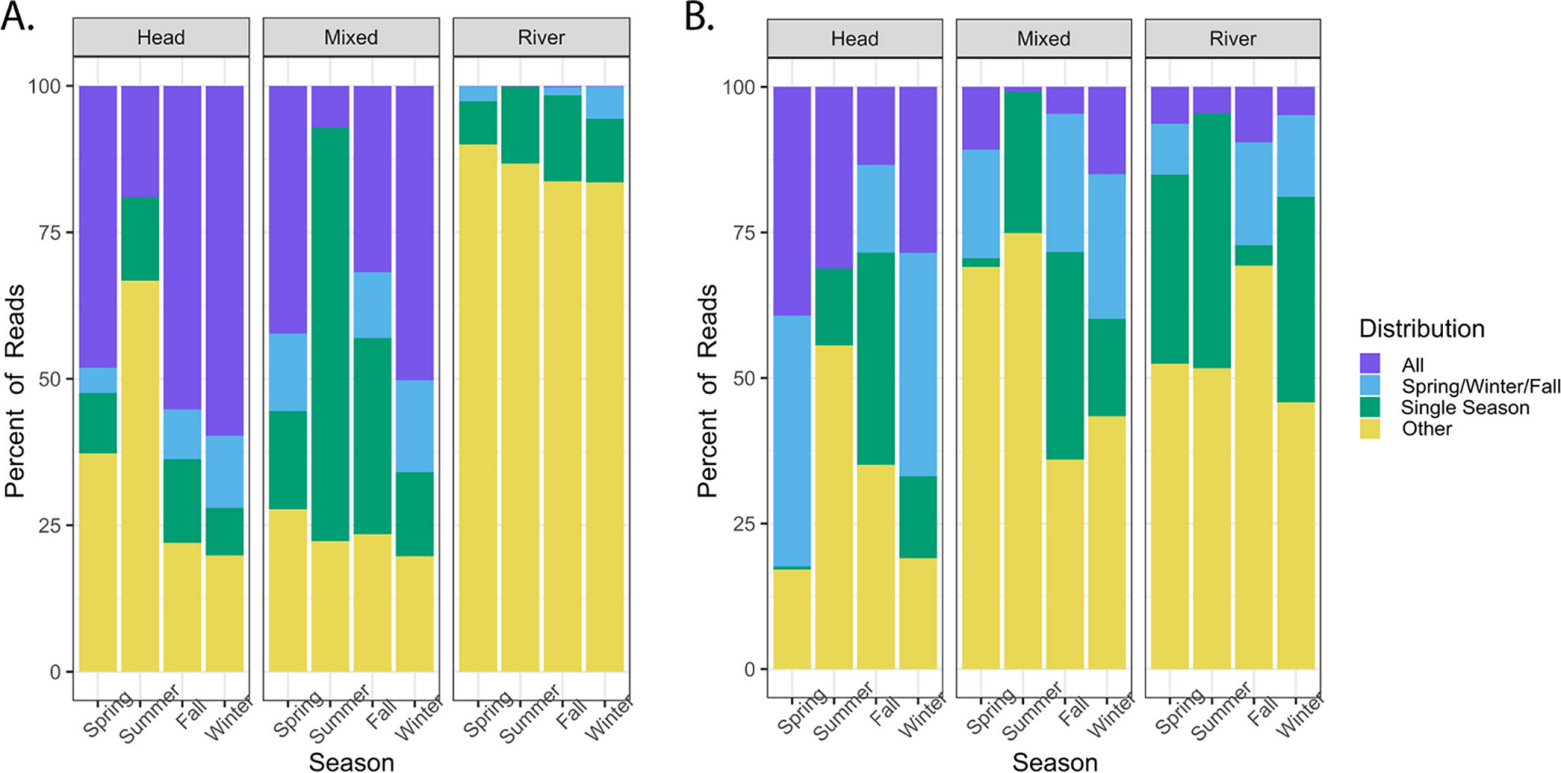
(A) Stacked bar plot showing the percentage of mappable reads in each sample belonging to ASVs in all seasons; ASVs in spring, winter, and fall; ASVs in a single season; and ASVs shared between two or three seasons (other). (B) Stacked bar plot showing the percentage of mappable reads in each sample belonging to viral contigs in all seasons; viral contigs in spring, winter, and fall; viral contigs in a single season; and viral contigs shared between two or three seasons (other).

Stacked bar plots based on the percentage of reads in each sample recruited to ASVs found in either a single site (head, mixed, and river) or shared among multiple sites (all, head/mixed, head/river, and mixed/river) were created to examine the relative abundance of unique and shared ASVs among sites (Fig. 2B). Despite the small number of ASVs shared among multiple sites (Fig. S1A), these shared ASVs contributed disproportionately to the communities, comprising between 25 and 80% of the total reads at each site (Fig. 2B). In all seasons except summer, the river had the largest number of reads mapping to ASVs unique to that site, while the head and mixed samples had more reads that mapped to ASVs shared between all three sites. In the spring, fall, and winter, the head and mixed sites shared more sequence reads than any other two sites. As reflected in the NMDS clustering (Fig. 2A), the summer samples were distinct from the other time points in that >50% of the reads were shared between the mixed and river sites, and even the head sample showed an increase in the abundance of ASVs shared with the river. Overall, the percentage of reads recruited to the few ASVs shared among all sites was highest in the head and decreased toward the river site. However, this percentage was the smallest (<5%) in the summer samples. Similar patterns were seen in bar plots based on the percentage of reads in each sample that recruited to ASVs found in either a single season (single), shared in all seasons (all), shared among all seasons excluding summer (spring/winter/fall), and all other combinations (other) (Fig. 3A). ASVs found in the all and spring/winter/fall categories made up 52 to 72% of the reads in the head samples, with the exception of the summer sample. The percentage of reads recruited to these categories decreased in the mixed samples (43 to 65%) and further decreased to 3 to 6% in the river samples, excluding the summer samples, which consistently had the lowest percentage of reads (0.1 to 19%) in these categories. Over 70% of the reads from the mixed summer samples were unique to that season, reflecting different dynamics in the summer than the other seasons (Fig. 2B and 3A).

**FIG 4:**
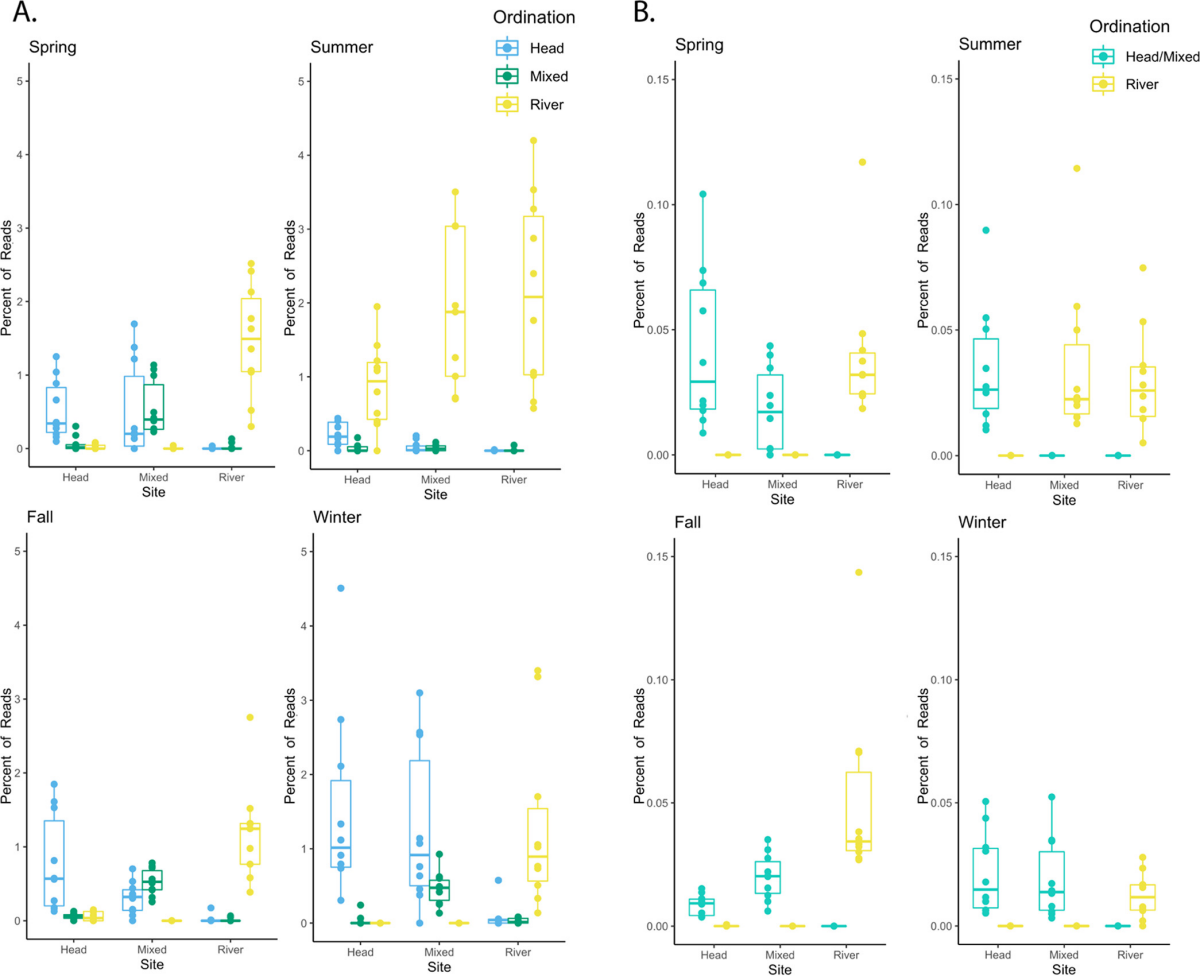
(A) Box-and-whisker plots representing the percentages of 16S rRNA gene sequences in each sample belonging to the 30 most influential ASVs significant to community ordination. (B) Box plots representing the percentages of sequences in each sample belonging to the 20 most influential viral contigs significant to community ordination. Colors represent the site where the viral contigs are significant based on ordination.

Approximately 250 ASVs were significantly (*P* < 0.01) correlated with the ordination of the prokaryotic communities based on relative abundance (Fig. 2A). These ASVs were ranked based on the magnitude of their influence on community ordination, and box plots of the relative abundance of the 10 most influential ASVs in each cluster (i.e., those with the greatest vector length) were created to depict their variation at each site in each season (Fig. 4A). For the spring, fall, and winter samples, the mean relative abundance of ASVs was highest in the site in which they were most influential, with the exception of the mixed site, where the ASVs associated with the head were more abundant in the spring and winter. Notably, in the summer samples, ASVs that were most influential in the river were the most abundant at all sites.

### Prokaryotic community composition based on taxonomy.

**FIG 5:**
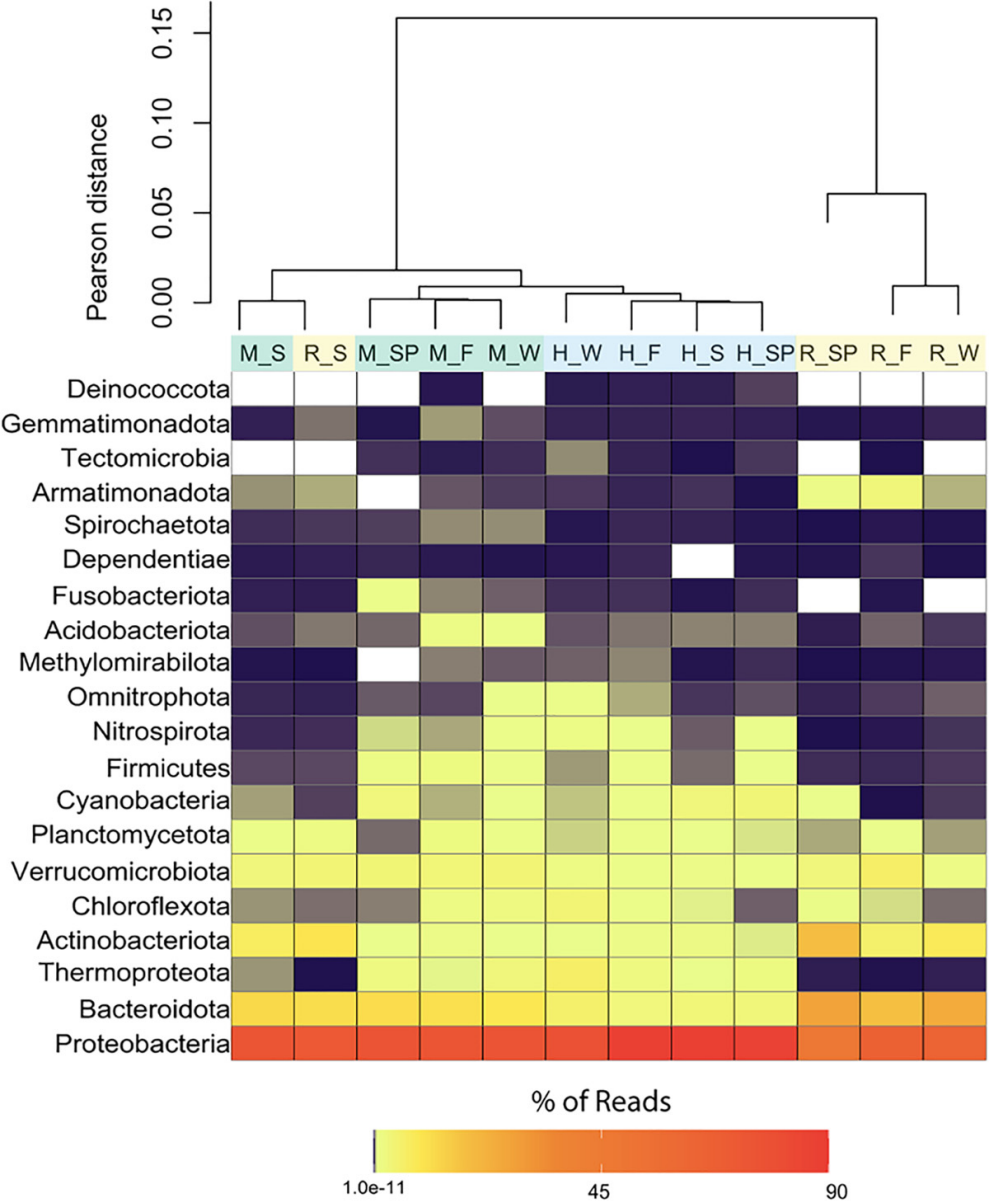
Heat map representing the percentages of 16S rRNA gene sequences in each sample belonging to the 20 most abundant phyla (which comprised >99% of total sequences for each sample). Dark orange indicates a higher percentage of sequence abundance, and white represents the absence of the phylum. The dendrogram was constructed using Pearson distance, and samples are colored based on site (blue, head; green, mixed; yellow, river). The first letter denotes the spring site (H, head; M, mixed; R, river). The letter(s) after the underscore represents season (F, fall; S, summer; SP, spring; W, winter).

ASVs were assigned taxonomy based on comparisons to the SILVA database, and names were adjusted to conform to Genome Taxonomy Database nomenclature (16). All samples were dominated by members of two phyla, *Proteobacteria* and *Bacteroidota*, which comprised between 75 and 91% of the sequences from each sample. A heatmap of the top 20 most abundant phyla, which encompassed >99% of all sequences in each sample, was constructed, including a dendrogram based on Pearson distances (Fig. 5). The head samples clustered together regardless of season. The river and mixed samples also clustered based on site, with the exception of their summer samples. The summer samples from the mixed and river sites formed their own cluster, which was more similar to the head and mixed samples than the river. With the exception of the summer river sample, which clustered with samples from the head and mixed sites, the river samples were the most taxonomically distinct, with a smaller proportion of reads from *Proteobacteria*, *Firmicutes*, *Nitrospirota*, *Fusobacteriota*, and *Tectomicrobia* and a larger proportion of reads belonging to *Bacteroidota*, *Thermoproteota*, and *Armatimonadota*.

### Viral community composition.

A total of 59,515 viral contigs were predicted using the Cenote-Taker 2 pipeline (17). An NMDS plot, with a stress value of 0.09, was constructed based on the Bray-Curtis dissimilarity of the relative abundances of the viral contigs in each sample (Fig. 2C). A significant positive correlation existed between the composition of the prokaryotic and viral communities (Mantel statistic, 0.2446; *P* = 0.001), with samples generally grouping by site in all seasons except summer. However, unlike the ASV analysis of prokaryotic communities, the NMDS based on viral contigs showed only two distinct clusters. One of the two viral clusters contained the river samples, and the other contained all the head and mixed samples, with the exception of the mixed summer sample, which grouped with the river. The statistically significant physiochemical parameters were mapped onto the NMDS and included ammonium, conductivity, DO, nitrate, nitrite, phosphate, and pH. The ordinal ellipses, representing the 95% confidence interval of the standard error, overlap for the mixed and head samples, but the ordinal ellipses for the river samples do not, suggesting distinct population means between the head/mixed cluster and the river cluster. A pairwise comparison confirmed the head and mixed viral communities were not significantly different. Similar to the prokaryotic communities, a two-way PERMANOVA demonstrated that season, site, and the interaction between them were significant to the ordination of the viral communities (*P* = 0.001, *R*^2^ = 0.21; and *P* = 0.001, *R*^2^ = 0.23, *R*^2^ = 0.33, respectively).

The distribution of viral contigs generally followed patterns similar to those observed for the prokaryotic ASVs. The majority of viral contigs were unique to one site, with ∼1% of viral contigs in the mixed and river sites and 7% of viral contigs in the head site shared across all seasons (Fig. S1B). Similar to the prokaryotic ASVs, very few viral contigs were shared among all seasons in a given site (Fig. S2B), and the spring head had more viral contigs in common with the mixed region (41 to 63%) than the river (0.3 to 1%) across all sampling time points, with the exception of summer (Fig. S1B). In the summer, the mixed and river sites shared the highest percentage of viral contigs (29.2%), compared to less than 5% for all other seasons.

A stacked bar plot based on the percentage of reads in each sample mapping to viral contigs found in either a single site (head, mixed, river) or shared among multiple sites (all, head/mixed, head/river, mixed/river) was distinct at each site (Fig. 2D). Less than 20% of the virome reads in each sample mapped to the viral contigs found in all sites. Similar to the prokaryotic communities, the majority (87 to 95%) of the mappable reads from the head samples mapped to viral contigs either found only in the head or shared between the head and mixed samples. Likewise, the majority (>90%) of the mappable reads from the river samples mapped to viral contigs found only in the river or shared between the mixed and river sites. More than half of the mappable reads in the mixed spring, fall, and winter samples were recruited to viral contigs found in both the head and mixed sites. In contrast, 75% of the mappable reads in the mixed summer sample recruited to viral contigs shared between the mixed and river sites. The head summer sample also had an increase in the percentage of reads belonging to viral contigs found in the head and river sites, further supporting the different dynamics occurring in summer. Similar to the prokaryotic community analysis, stacked bar plots based on the percentage of reads in each sample that recruited to viral contigs shared among seasons revealed the percentage of reads recruiting to the all and spring/winter/fall categories was highest in the head (28 to 82%) and decreased in the mixed (1 to 59%) and river samples (4 to 27%) (Fig. 3B).

Approximately 800 viral contigs were significantly (*P* < 0.01) correlated with the ordination of the viral communities based on relative abundance. These viral contigs were ranked based on the magnitude of their influence on community ordination, and box plots depicting the relative abundance of the 10 most influential viral contigs in each cluster (based on vector lengths) were made to depict their variation at each site and in each season (Fig. 4B). In contrast to the prokaryotic community, viruses from the mixed and head samples did not form distinct clusters on the NMDS (Fig. 2C) and instead were represented together. In the spring, fall, and winter samples, the mean relative abundance of viral contigs was highest in the sites in which they were most influential (Fig. 4B). However, in the summer samples, viral contigs that were influential in the river were the most abundant in both the river and mixed sites. At the contig level, the viral communities in the summer mixed site appeared more similar to the river sites.

### Viral community composition at a finer resolution.

**FIG 6:**
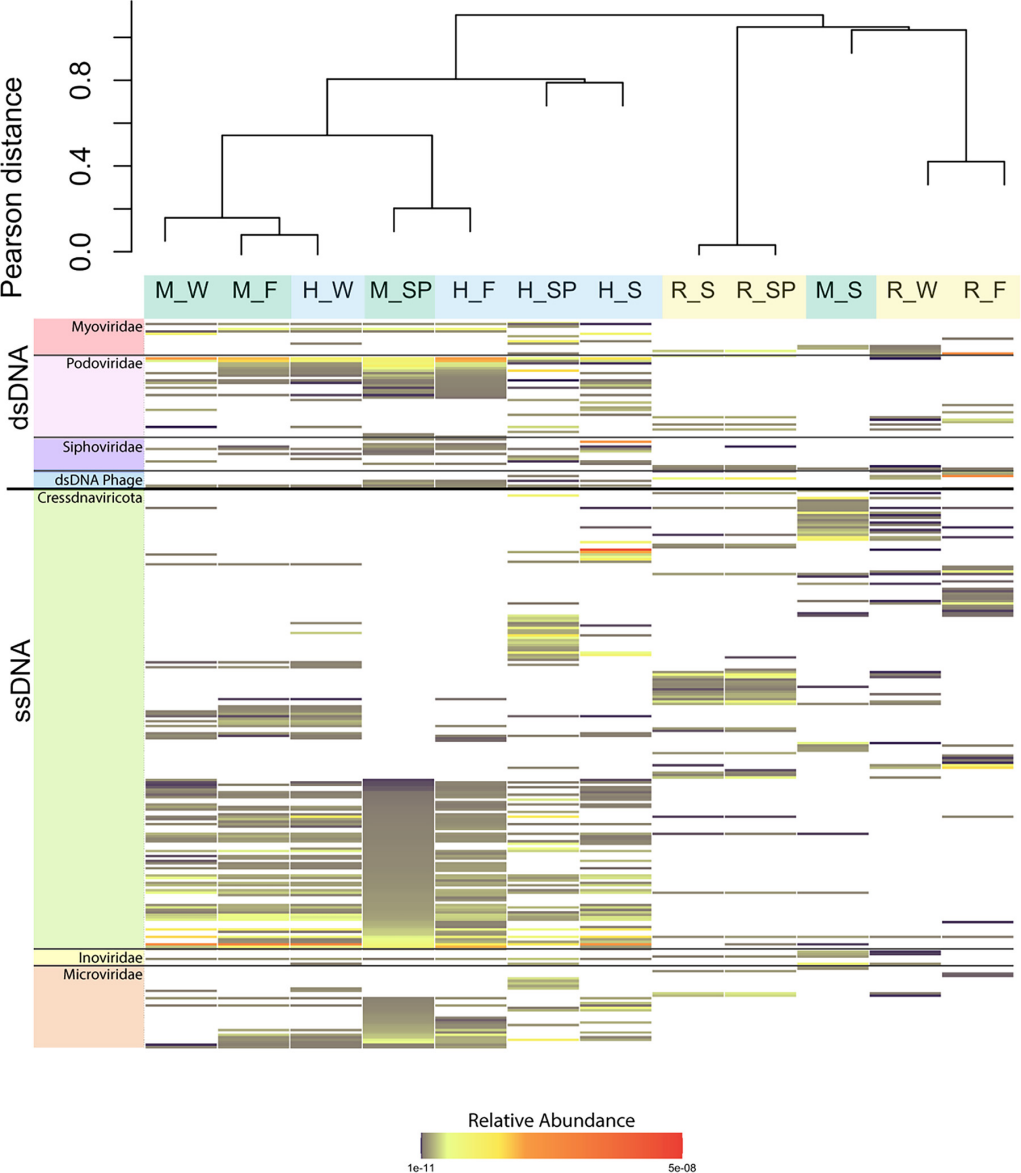
Heat map representing the relative abundance of viral sequences in each sample belonging to each of the complete viral genomes. Dark orange indicates a higher sequence abundance, and white represents the absence of the phylum. The dendrogram was constructed using Pearson distance, and samples are colored based on site (blue, head; green, mixed; yellow, river). The first letter denotes the spring site (H, head; M, mixed; R, river. The letter(s) after the underscore represents season (F, fall; S, summer; SP, spring; W, winter). Genomes are organized based on taxonomy.

The viral contigs were further curated to identify putative complete genomes, which included 227 single-stranded DNA (ssDNA) virus genomes and 69 double-stranded DNA (dsDNA) phage genomes (Table S2). Heatmaps were constructed using the relative abundance of each complete circular viral genome in each sample (calculated as read coverage normalized by genome length and library size), with a dendrogram based on hierarchical clustering (Fig. 6). Analysis of complete viral genomes alone recapitulated the patterns observed with the viral contig analysis, with two main clusters, one containing the river samples and the mixed summer sample and the other containing the head samples and the mixed samples from other seasons.

Eukaryotic viruses from the phylum *Cressdnaviricota* (18) dominated the complete ssDNA virus genomes, which also included members from the two major groups of ssDNA phage (35 genomes from the family *Microviridae* and 5 genomes from the family *Inoviridae*). All of these ssDNA viruses have been increasingly recognized for their ubiquitous presence in aquatic ecosystems, although their hosts and ecological roles are largely unknown (19–23). Overall, the ssDNA viruses were more abundant in the head and mixed samples than in the river samples. This trend was most noticeable for the *Microviridae*, where only 5 of the 45 complete genomes were found in the river.

Phylogenomic analysis of the 69 dsDNA phage genomes belonging to the order *Caudovirales* with the GL-UVAB (genomic lineages of uncultured viruses of *Archaea* and *Bacteria*) tool (24) demonstrated that these sequences belong to 19 viral lineages, including two previously undescribed lineages that represent novel phage diversity recovered from this lotic system (Fig. S3A). A stacked bar chart of the percentage of reads mapping to genomes in each lineage showed the temporal variation of phage lineages in all sites, although the head and river sites showed more consistency than the mixed site (Fig. S3B). The mixed samples were the most variable, sharing only a single lineage among all four seasons. As seen in the other analyses, the mixed samples shared the most lineages with the head samples in all seasons except summer, when the relative abundance of viral lineages in the mixed and river samples were nearly identical. Using CRISPR spacers, shared tRNAs, and sequence homology, host predictions were performed for each dsDNA phage genome assigned to a lineage to analyze the relative abundance of viral contigs predicted to infect different host phyla and families (Fig. S4). Predicted hosts included members of *Proteobacteria*, *Bacteroidota*, *Firmicutes*, *Fusobacteriota*, and *Actinobacterota* phyla. The taxonomy of the predicted hosts aligned with the prokaryotic community analysis, with the top 20 most abundant phyla identified by 16S rRNA gene sequencing well represented in the predicted hosts for the dsDNA phage (Fig. 3 and Fig. S4A).

## DISCUSSION

### Prokaryotic and viral communities follow similar patterns over the spring-river continuum.

Prokaryotic cell and VLP abundances increased with distance from the head of the spring. The 100-fold increase in prokaryotic and VLP abundances from the head to the river site corresponds to changes in physiochemical parameters, such as increasing DO, phosphate, ammonium, and nitrite concentrations (see Table S1 in the supplemental material). Prokaryotic cell and viral abundances measured in the river in this study are on the lower end of previously reported concentrations in other rivers (25–28), likely due to the significant influence of groundwater in the lower Suwannee River (8). The groundwater supplying the spring head is derived from the Floridan aquifer, which is devoid of sunlight and poor in organic carbon sources (29). Exposure to sunlight and the addition of organic carbon from the surrounding environment could lead to an increase in microbial abundances as water flows through the system, supplemented by the introduction of prokaryotes and viruses from the river upstream or terrestrial environment through runoff and leaf litter (30). The area surrounding the spring has been partially developed for agricultural and urban use (∼55%), while the rest is comprised of natural forest and wetland (15).

Along with cell and VLP abundance increasing with distance from the spring head, the composition of prokaryotic and viral communities was different at discrete locations in the Manatee Springs system. The NMDS plots of prokaryotic and viral community composition both showed a general grouping of samples by site in all seasons except summer. However, one major difference is that the prokaryotic communities formed three distinct clusters (one for each site), while the viral communities only contained two clusters (head and mixed sites overlap, river is distinct). The greater overlap between the head and mixed sites could be attributed to a delayed response from the viral community. As the viral and prokaryotic communities converge, changes in specific host densities could hinder viral reproduction (31), inhibiting the formation of a distinct viral community in the mixed site.

### Distinct community dynamics in the summer.

The prokaryotic and viral communities generally clustered by site, with the notable exception of the mixed samples in summer, which clustered with the river samples. These patterns were observed at multiple levels of resolution, including full community NMDS analyses based on relative abundance of all ASVs and viral contigs (Fig. 2A and C, respectively), the relative abundance of individual ASVs and viral contigs (Fig. 4), and the taxonomy of ASVs (Fig. 5) and complete viral genomes (Fig. 6 and Fig. S3).

The clustering patterns of the samples can be best explained in the context of the hydrology of the Manatee Springs system. Flow of groundwater out of the spring is dependent on the differential between the aquifer level (the spring’s source) and the Suwannee River. Under dry conditions, when the water level is higher in the aquifer at the spring head than in the river, water flows from the aquifer into the river (32). However, during large rain events, river water levels are impacted more dramatically and rapidly than aquifer water levels, which can lead to a brief period where the gradient temporarily slows or the system flow reverses (33, 34). Spring discharge rates from each collection date reported by the United States Geological Survey’s continuous water monitoring data show that the summer collection had the lowest spring discharge rate and the highest river flow rate (Table S1). While the spring flow did not reverse, the higher river flow combined with the lower spring flow rate during the rainy season likely allowed for intrusion of river water into the spring run (the mixed site), driving the similarities between the mixed and river sites during the summer season for both the prokaryotic and viral communities. With less discharge from the spring head in the summer, the river had a greater influence on the mixed site as opposed to being dominated by flow from the head communities. This idea is reinforced by the similarity in conductivity values seen at the mixed and river sites during the summer sampling, which reflected a shift from the values seen during the other sampling dates (Table S1), as well as changes in the relative abundance of the most influential ASVs and viral contigs in the summer season (Fig. 4).

### Analysis of spring microbial communities through a metacommunity lens.

Aquatic microbial ecology studies are increasingly including metacommunity theory, which considers dynamic processes occurring at different spatial scales and how these processes affect communities linked by dispersal of their potentially interacting component species, to understand microbial community structure (35). To our knowledge, no prior studies have simultaneously examined both prokaryotic and viral communities in freshwater ecosystems through the lens of metacommunity theory (36, 37). Efforts to understand prokaryotic dynamics in lotic systems within a metacommunity framework have identified both spatial and temporal factors affecting community structure, with community assembly primarily attributed to either species sorting or mass effects (5, 38). In communities influenced by species sorting, local environmental conditions determine community structure, which is relevant to the Manatee Springs system as environmental conditions change rapidly as water flows through the system. However, the low residence time of communities in fast-flowing systems, such as Manatee Springs, may lessen the influence of local environmental factors. Systems with low residence times are expected to be more influenced by mass effects (in which high rates of dispersal play a greater role than adaptation) due to the high input of microbes (39). Similar to prior work in other lotic systems, evidence of species sorting and mass effects were both observed in the prokaryotic and viral communities in this study. However, the influence of each paradigm varied by the community and site being examined.

Species sorting appeared to play a large role in shaping the prokaryotic communities and, to a lesser extent, the viral communities of the Manatee Springs system. Despite the low residence time of the system, only a small portion of ASVs and viral contigs were shared among all sites in each season (Fig. S1A). These few shared ASVs and viral contigs comprised a large percentage of the sequences in the head samples; however, this percentage decreased in the river, suggesting that these members do not persist in large abundances downstream (Fig. 2B). Distinct prokaryotic community structures were observed for each of the three sites within Manatee Springs (Fig. 2A). Additionally, taxonomic differences were seen within these communities, suggesting a selective influence of environmental conditions. For example, members of the phyla *Firmicutes* and *Thermoproteota* comprised a larger percentage of the sequences in samples from the head site but were virtually absent in the river site (Fig. 5). While it is difficult to assess metabolic functionality at the phylum level, these results are consistent with known features of these phyla. *Firmicutes* are typically spore-forming bacteria that may persist in the aquifer; some members are also anaerobic and capable of living in extreme environments (40). Likewise, most members of the phylum *Thermoproteota* are chemolithoautotrophic ammonia oxidizers well suited to function under the low-oxygen and oligotrophic conditions of the spring head (41). Overall, these findings suggest that the prokaryotic community assemblages in the head, mixed, and, potentially, river site are being driven by species sorting.

Similar to the prokaryotic communities, there is evidence of species sorting in the viral communities. Few viral community members are shared among the sites in all seasons (Fig. S1B), and the ones that are are found in low abundance (Fig. 2D). When looking at the complete virus genomes, most genomes found in the head site were not present in the river site, potentially pointing to selection (Fig. 6). However, the major difference seen between the prokaryotic and viral communities is that the viral communities do not exhibit unique community structure at all three sites. The community structures for the head and mixed sites exhibited great overlap (Fig. 2C). Viruses are obligate parasites dependent on their hosts for replication. As suggested above, shifts in the host population likely delay viral response to environmental changes. As a result, the viral communities are more susceptible to being overcome by high dispersal rates and, therefore, are more heavily influenced by mass effects than the prokaryotic communities. This is most evident in the viral communities from the mixed site, which fail to form a cluster distinct from the head, demonstrating the vulnerability of this site to shifts in hydrology.

Overall, both species sorting and mass effects play a role in structuring prokaryotic and viral communities within the Manatee Springs system. While the presence of ASVs and viral contigs shared between all sites indicated that these microbial communities were carried downstream by the spring’s swift flow, the abundance data suggested that the large groundwater input only played a minor role in seeding communities downstream. This could be for one of two reasons. It could be that the drastic changes in environmental parameters outside the aquifer, such as UV exposure and higher oxygen concentrations, select against members of the head communities remaining dominant once introduced to an exposed lotic system, where they must compete against taxa that can readily take advantage of abundant resources in the river. It could also be that groundwater-associated prokaryotic and viral communities were largely overtaken by local communities in the river, which were present at 100-fold higher concentrations. More than likely, both species sorting and mass effects play a role in microbial community structure in this lotic system, with the most influential paradigm varying for different communities and sites.

### Greater temporal stability in the spring head than in the downstream sites.

Although both the prokaryotic and viral communities exhibited temporal variability, especially in the summer, the composition of these communities fluctuated less in the spring head than in the two other downstream sites, likely due to the stability of the groundwater environment. Our previous work compared prokaryotic and viral communities from the heads of five different springs fed by the FAS (Ichetucknee, Jackson, Manatee, Rainbow, and Volusia). The study concluded that each spring harbored unique prokaryotic and viral communities; however, samples were only collected at a single time point (15). The present study expanded on this work by examining multiple seasons and locations within Manatee Springs. Comparing the present data to the prior results from other first-magnitude springs in an NMDS plot further supported the distinct microbial communities in each spring system, with the Manatee Springs head samples clustering away from the head samples from other springs regardless of season (Fig. S5 and S6). Therefore, while the prokaryotic and viral communities within Manatee Springs vary temporally, these communities remain distinct from other springs fed by the same aquifer.

### Conclusions.

Baseline information regarding the spatiotemporal dynamics of microbial communities in river systems fed by springs is needed to examine community assembly mechanisms and predict future changes in these understudied lotic systems. The large groundwater input from the springs plays a major role in influencing downstream conditions in river systems, impacting nutrient concentrations and potentially introducing novel groundwater prokaryotes and viruses to the rivers. While flowing through this system, microbial communities experience changing physiochemical conditions and the introduction of novel members from the surrounding environment. In this study, of a lotic environment fed by the Floridan aquifer, both the location within the system and the season had significant effects on the composition of the prokaryotic and viral communities. In particular, the lower discharge rates from the spring head in the summer led to a greater influence of the river on the mixed site in summer, compared to the other seasons where the mixed site was dominated by flow from the head communities. Throughout the seasons, the greater abundance of persistent members in the spring head indicated that this site was more stable than downstream sites, and comparisons to prior work reaffirm that distinct first-magnitude springs maintain unique prokaryotic and viral communities despite seasonal variation. While some microbes from the spring head persisted in the river, they were present in low abundance, suggesting that the springs play only a minor role in seeding downstream viral and prokaryotic communities. Through the framework of metacommunity theory and community coalescence, both species sorting and mass effects have an impact on microbial communities of this lotic system. Further work over longer time scales is needed to parse the exact impacts of these mechanisms on community structure. However, this work establishes a foundational understanding of the composition and variability of prokaryotic and viral communities of these important freshwater ecosystems.

## MATERIALS AND METHODS

### Sample collection and processing.

This study investigated the spatiotemporal dynamics of prokaryotic and viral communities from three sites in the Manatee Springs system (29.50°N 82.98°W), a first-magnitude spring in northwestern Florida (Fig. 1). The first site (the spring head) is the spring’s outflow, the second site (the mixed region) is where the spring water mixes with the water from the Suwannee River, and the final sampling site (the river site) is located in the Suwannee River approximately ∼400 m downstream of the spring outlet.

Fifty-liter samples were collected at the spring head as close to the spring outflow as possible using a 5-liter horizontal PVC water sampler (Forestry Suppliers), and 25-liter water samples were collected from just below the surface of both the mixed and river sites. Each site was sampled in triplicate on four sampling dates (6 May 2017, 12 July 2017, 26 October 2017, and 19 January 2018), selected to correspond with the seasons (spring, summer, fall, and winter, respectively). However, northwestern Florida’s climate mainly consists of two seasons distinguished by rainfall, with May through October broadly constituting the rainy season (42). The July and January samples therefore represent the rainy and dry season, respectively, while the May and October samples represent the transitional periods between the two.

Relevant environmental parameters were measured at each sampling event. Temperature, pH, turbidity, DO, and conductivity were measured using a YSI Pro DSS handheld multiparameter water quality meter. Spring and river discharge rates from each collection date were obtained from the United States Geological Survey’s continuous water monitoring data for Manatee Springs (https://waterdata.usgs.gov/usa/nwis/uv?site_no=02323566) along with precipitation measurements for the area. Ammonium, nitrite, nitrate, phosphate, and silicate analyses were performed with a Lachat QuickChem 8500 Series 2 system (see Table S1 in the supplemental material). Additionally, triplicate 100-ml water samples were collected and fixed with paraformaldehyde (final concentration, 2%) for determining the abundance of prokaryotic cells and VLPs by SYBR Gold staining and epifluorescence microscopy (43). All samples were transported back to the lab within 4 h of collection for processing by following the procedures detailed by Malki et al. (15) and summarized below. Briefly, samples were concentrated using a 30-kDa tangential flow filter (TFF; GE Healthcare) from 50 liters (head) or 25 liters (mixed and river) of water to ∼200 ml, and the concentrates were subsequently filtered through Sterivex filters (0.22-μm pore size). Prokaryotic DNA was extracted from the Sterivex filters, while the filtrate was further processed to generate viromes.

### 16S rRNA gene amplicon sequencing.

DNA was extracted from the Sterivex filters using the MoBio PowerSoil DNA isolation kit, and the V4 hypervariable region of the 16S rRNA gene was amplified using primers 515f and 806r (44, 45). Approximately 20,000,000 paired reads were generated using the Illumina MiSeq platform by the Michigan State University Research Technology Genomics Core facility. Processing and analysis of sequence data were conducted using R with RStudio version 1.0.153 (46, 47). Sequences were trimmed using Trimmomatic v 0.36.0 (48) and processed using the Divisive Amplicon Denoising Algorithm (*DADA2*) package v 1.6.0 in RStudio (49), generating amplicon sequence variants (ASVs). The ASVs were then compared against the SILVA v 132 rRNA database for taxonomic assignments (default bootstrapping value of 50) (50). The data were normalized based on library size using the *DESeq2* v 1.18.1 package in RStudio (51). Rarefaction curves demonstrated that all samples reached asymptotes, indicating adequate sequence coverage (data not shown).

### Virome preparation and sequencing.

Viral particles were obtained from the Sterivex-filtered TFF concentrates described above. The particles were further concentrated by incubating overnight with 10% (wt/vol) polyethylene glycol 8000 at 4°C, followed by centrifugation at 11,000 × *g* for 45 min. The pellets containing viral particles were resuspended in 1 ml of 0.02-μm-filtered water from their respective sampling sites and treated with 20% chloroform and 10 U of Turbo DNase (Thermo Fisher) per milliliter of sample for 30 min to remove remaining cells, vesicles, and free DNA (15, 52, 53). Qiagen’s QIAamp MinElute virus spin kit was used to extract DNA from the purified viral particles. Samples were fragmented to 300 bp using a Covaris M220 instrument, processed with the Accel-NGS 1S plus DNA library kit for Illumina Platforms (Swift Biosciences), and sequenced using a high-output V2 kit (300 cycle) on a NovaSeq 6000 platform at the University of Colorado BioFrontiers Next-Gen Sequencing Core Facility.

Approximately 1.2 billion viral metagenomic paired reads were generated and processed on the University of South Florida high-performance computing cluster. Adapters and low-quality reads were removed from sequences using Trimmomatic v 0.36.0 (48) with default parameters, with the exception of a 10-bp headcrop. Trimmed sequences were quality controlled using FastQC v 0.11.5 (54), and assemblies were performed using metaSPAdes v 3.11.1 with default parameters (55). Assembled contigs were filtered by size on the Galaxy web-based platform, retaining only contigs larger than 1 kb, which were then processed with the Cenote-Taker 2 pipeline (beta version, GitHub commit 85fa905977e83ad9f7566a31f1a75d1e259fb6b8, HMM database version 1.0 [8 February 2020; https://zenodo.org/record/3659320]). The Cenote-Taker 2 pipeline was used for prediction and taxonomic assignment of both linear and circular viral sequences (here referred to as viral contigs) (56, 57).

A nonredundant contig file was created using RedRed (https://github.com/kseniaarkhipova/RedRed). Trimmed forward reads from each sample were mapped to the nonredundant file of viral contigs using Bowtiebatch v 1.0.1 and Read2RefMapper v 1.0.1 applications in the iVirus pipeline (58, 59). Reads were mapped with at least 90% identity and viral contigs were considered present if at least 75% of the contig length was covered (60). The number of reads mapping to a given contig was normalized by contig length and sequence library size. In the viral analysis, two samples (one replicate from the summer mixed sample and one replicate from the fall mixed sample) were excluded from all downstream analyses due to likely mislabeling during sequencing. Therefore, both the summer and fall mixed samples have only two replicates instead of three.

### Prokaryotic and viral community structure analysis.

Prokaryotic and viral communities were compared based on both the presence and absence, as well as the relative abundance, of ASVs and viral contigs, respectively. Venn diagrams depicting both the ASVs and viral contigs shared among sites in each season were constructed using the vennDiagram function in the *Limma* v 3.44.3 package (61). NMDS plots based on a Bray-Curtis dissimilarity of the relative abundance of ASVs and viral contigs were constructed using the metaMDS function in *vegan* v2.3-1 with ordinal ellipses representing the 95% confidence interval of the standard error, and the envfit function was used to map statistically significant parameters (62). The adonis2 function was then used to calculate a two-way PERMANOVA using both site and collection date. A Mantel test to determine the correlation of the prokaryotic and viral communities was performed in *vegan* v 2.3-1 using the Mantel function.

Stacked bar plots based on the percentage of reads in each prokaryotic and viral sample recruited to ASVs and viral contigs, respectively, found in either a single site (head, mixed, or river) or shared among multiple sites (all, head/mixed, head/river, or mixed/river) were made in *ggplot2* (63). Overlapping ASVs and viral contigs were extracted using the calculate.overlap function in the *VennDiagram* v 1.6.20 package and were parsed from the original counts table using the subset function in *phyloseq* v 1.22.3 (64). This process was repeated to create a stacked bar plot based on the percentage of reads in each sample belonging to ASVs and viral contigs found in either a single season (single season), shared among all seasons (all), shared among spring, fall, and winter (spring/winter/fall), or shared among other seasons (other).

### Phylum-level analysis of the prokaryotic communities.

Prokaryotic sample replicates were then merged using the *phyloseq* v 1.22.3 package. A heatmap based on the relative abundance of the top 20 phyla, ranked across all samples, was constructed using the *Superheat* package v 1.0.0 along with a dendrogram based on the Pearson distance calculated using the cor function in R (65, 66). A stacked bar plot based on the percentage of sequences in each sample belonging to ASVs found in either a single site (head, mixed, river) or shared among multiple sites (all, head/mixed, head/river, mixed/river) was constructed in *ggplot2* (63). Overlapping ASV names were extracted using the calculate.overlap function in the *VennDiagram* v 1.6.20 package and were parsed from the original counts table using the subset function in *phyloseq* v 1.22.3 (64). This process was repeated to create a stacked bar plot based on the percentage of reads in each sample belonging to ASVs found in either a single season (single season), shared among all seasons (all), shared among spring, fall, and winter (spring/winter/fall), or shared among other seasons (other).

### Analysis of complete circular viral genomes.

The viral contigs identified by the Cenote-Taker 2 pipeline underwent further manual curation to identify complete genomes (57). Viral contigs were considered complete genomes (Table S2) if they were within the size range of their assigned viral families as defined by the International Committee on the Taxonomy of Viruses (ICTV) and contained the hallmark genes of their respective families (67). As outlined above, read mapping was used to compare the distribution and abundance of each of these genomes in each sample (values were averaged between replicates). The *Superheat* v 1.0.0 package was used to create a heatmap of genome coverage paired with a dendrogram based on Pearson distance calculated using the cor function in R (65, 66). The complete dsDNA phage genomes identified by the pipeline were sorted into genomic lineages using a phylogenomic approach with available genomes in the GL-UVAB reference database (24). A tree depicting the viral lineages represented in this study was constructed using Dice distances and neighbor-joining as described in Coutinho et al. (68). Briefly, viral sequences were clustered using CD-Hit with a 95% nucleotide identity cutoff and a minimum 50% coverage to remove redundant sequences, distances were calculated based on a modified Dice method, and an all-versus-all comparison was performed using Diamond (69, 70). Lineages were assigned based on node depth cutoffs established in Coutinho et al. (68). Stacked bar plots of the relative abundance of the dsDNA phage, broken down by viral lineage, were constructed using the package *ggplot2* (63). Note that this pipeline was trained with dsDNA phage genomes; thus, ssDNA phage sequences were not included in this analysis and will instead be explored in more detail in a future manuscript.

### Analysis of significant ASVs and viral contigs.

The relative abundance of ASVs and viral contigs significant to the ordination of the prokaryotic and viral NMDS, respectively, were selected based on their influence for each cluster. All significant ASVs were determined using the envfit function in *vegan* v 2.3-1 (62). The significant ASVs were then ranked based on the length of the vectors, with greater length corresponding to greater influence. The relative abundance of the top 10 most influential, significant ASVs for each cluster were then plotted in a box-and-whisker plot in *ggplot2* (63). The box plot depicts the relative abundance of the 30 most influential ASVs at each site through each season, with color indicating their cluster affiliation and each individual point representing an ASV.

This analysis was repeated with viral contigs with a small variation. The significant ASVs found in the prokaryotic analysis were all within the top 10% of ASVs ranked based on abundance across samples. To enable the analysis of the much larger viral data set, the viral contigs were also ranked based on their relative abundance across samples and subset by the top 10% most abundant viral contigs. Significant viral contigs were then determined by analyzing these 6,000 viral contigs using the envfit function in *vegan* v 2.3-1 (62). The most influential viral contigs were again found by ranking based on vector length. The relative abundance of the top 10 most influential, significant contigs for each of the two clusters were then plotted in a box and whisker plot in *ggplot2* (63). The box plot depicts the relative abundance of the 20 influential viral contigs in each site throughout the seasons, with color indicating their cluster affiliation and each individual point representing a contig.

### Computational viral host prediction.

Viruses classified within genome lineages were assigned candidate hosts using computational approaches that have been previously benchmarked (71). To this end, a previously described methodology with predefined cutoffs was applied (68). NCBI RefSeq genomes of *Bacteria* and *Archaea* were used as the reference database of potential hosts. Searches for CRISPR spacers, shared tRNAs, and sequence homology were performed as follows. CRISPR arrays were identified among reference prokaryote genomes using a custom script (72). Obtained CRISPR spacers were queried against the viral sequences using BLASTn (73). The cutoffs defined for these searches were a minimum identity of 80%, minimum query coverage of 100%, maximum of 1 mismatch, and maximum E value of 1. tRNAs were identified among viral scaffolds using tRNAScan-SE (74). Viral tRNAs were then queried against the database of reference prokaryote genomes using BLASTn. The cutoffs defined for these searches were a minimum alignment length of 60 bp, minimum identity of 90%, minimum query coverage of 95%, maximum of 10 mismatches, and maximum E value of 0.001. Finally, for sequence homology, viral sequences were queried directly against the database of reference prokaryote genomes through BLASTn. The cutoffs defined for these searches were a minimum alignment length of 300 bp, minimum identity of 50%, and maximum E value of 0.001. Once all three metrics of virus-host associations were computed, a consensus score was calculated for each virus-taxon pair with the following criteria: 3 points were added to the taxon if it was a CRISPR match, 2 points if it was a homology match, and 1 point if it was a shared tRNA. The taxon that displayed the highest score was defined as the putative virus host. Stacked bar plots of the relative abundance of the dsDNA phage, broken down by predicted host, were constructed using the package *ggplot2* (63).

### Comparison of prokaryotic and viral community composition of multiple springs.

16S rRNA gene data from both this study and the previous spatial study were processed using the Divisive Amplicon Denoising Algorithm (*DADA2*) package v 1.6.0 in RStudio (49), generating ASVs. The ASV tables from both studies were merged using the mergeSequenceTables function and normalized based on library size using the *DESeq2* v 1.18.1 package in RStudio (51). The Manatee Springs sample from the spatial study and the head spring sample from this study were from the same DNA extraction but sequenced separately. Viral contigs from both this study and the spatial study were merged and dereplicated using RedRed. Trimmed forward reads from each sample were mapped to the nonredundant file of viral contigs using Bowtiebatch v 1.0.1 and Read2RefMapper v 1.0.1 applications in the iVirus pipeline (58, 59). Reads were mapped with at least 90% identity, and viral contigs were considered present if at least 75% of the contig length was covered (60). The number of reads mapping to a given contig was normalized by contig length and sequence library size. The Manatee Springs viral sample from the spatial study and the head spring sample from this study are identical. NMDS plots based on a Bray-Curtis dissimilarity of the relative abundance of ASVs and viral contigs were constructed using the metaMDS function in *vegan* v 2.3-1 (62).

### Data availability.

Sequences are available at the NCBI Sequence Read Archive database (study number PRJNA666620, BioSample accession numbers SAMN16331665 to SAMN16331700). Virome raw sequences are available at the NCBI Sequence Read Archive database (study number PRJNA666620, BioSample accession numbers SAMN16363521 to SAMN16363556).
