# Supplementary material for: Spatial and Temporal Dynamics of Prokaryotic and Viral Community Assemblages in a Lotic System (Manatee Springs, Florida)
Source: Appl Environ Microbiol. 2021 Aug 26;87(18):e00646-21. doi: 10.1128/AEM.00646-21 (PMC8388828; doi:10.1128/AEM.00646-21)
Supplement: Supplemental file 1 — Tables S1 and S2, Fig. S1 to S6. Download AEM.00646-21-s0001.pdf, PDF file, 1.3 MB [file aem.00646-21-s0001.pdf]

Supplemental Table 1

Site	Head	Head	Head	Head	Mixed	Mixed	Mixed	Mixed	River	River	River	River
Season	Spring	Summer	Fall	Winter	Spring	Summer	Fall	Winter	Spring	Summer	Fall	Winter
Collection Date	6 May 2017	12 July 2017	26 October 2017	19 January 2018	6 May 2017	12 July 2017	26 October 2017	19 January 2018	6 May 2017	12 July 2017	26 October 2017	19 January 2018
Temp (ºC)	22.4	22.5	22.3	22.3	22.2	29.9	22.4	22.3	23	27.3	22.5	14.5
pH	7.3	7.5	6.9	7.3	7.4	7.5	7.0	7.4	8.0	7.7	7.2	7.7
Turbidity (NTU)	3.5	3.2	16.5	1.0	3.5	3.5	22.7	1.0	4.9	5.5	22.7	1.8
%DO	14.0	30.1	21.5	23.0	38.0	57.0	34.5	36.9	88.7	95.6	90.1	98.2
Conductivity (µS/cm)	504	508	547	534	501	490	547	534	378	313	312	355
Ammonium (µM)	0.12	0.37	0.93	1.13	2.26	0.79	0.54	1.50	3.47	1.63	2.39	2.19
Nitrite (µM)	0.04	0.14	0.03	0.02	0.13	0.21	0.20	0.06	0.30	0.44	0.74	0.34
Nitrate (µM)	153.1	165.3	63.4	175.0	151.7	174.0	189.1	173.6	100.7	87.4	191.6	92.3
Phosphate (µM)	0.56	0.16	0.72	0.70	0.42	0.2	0.68	0.71	1.3	1.64	2.59	2.26
Silicate (µM)	105.2	104.3	128.2	101.2	105.0	103.5	99.4	106.1	118.0	129.4	100.2	141.6
Discharge (ft^3/sec)	142.5	127.0	186.5	147.5	142.5	127.0	186.5	147.5	142.5	127.0	186.5	147.5

Supplemental Table 1. Sample collection dates and physiochemical parameters for each sample (NTU = nephelometric turbidity units, DO=dissolved oxygen).

Supplemental Figure 1

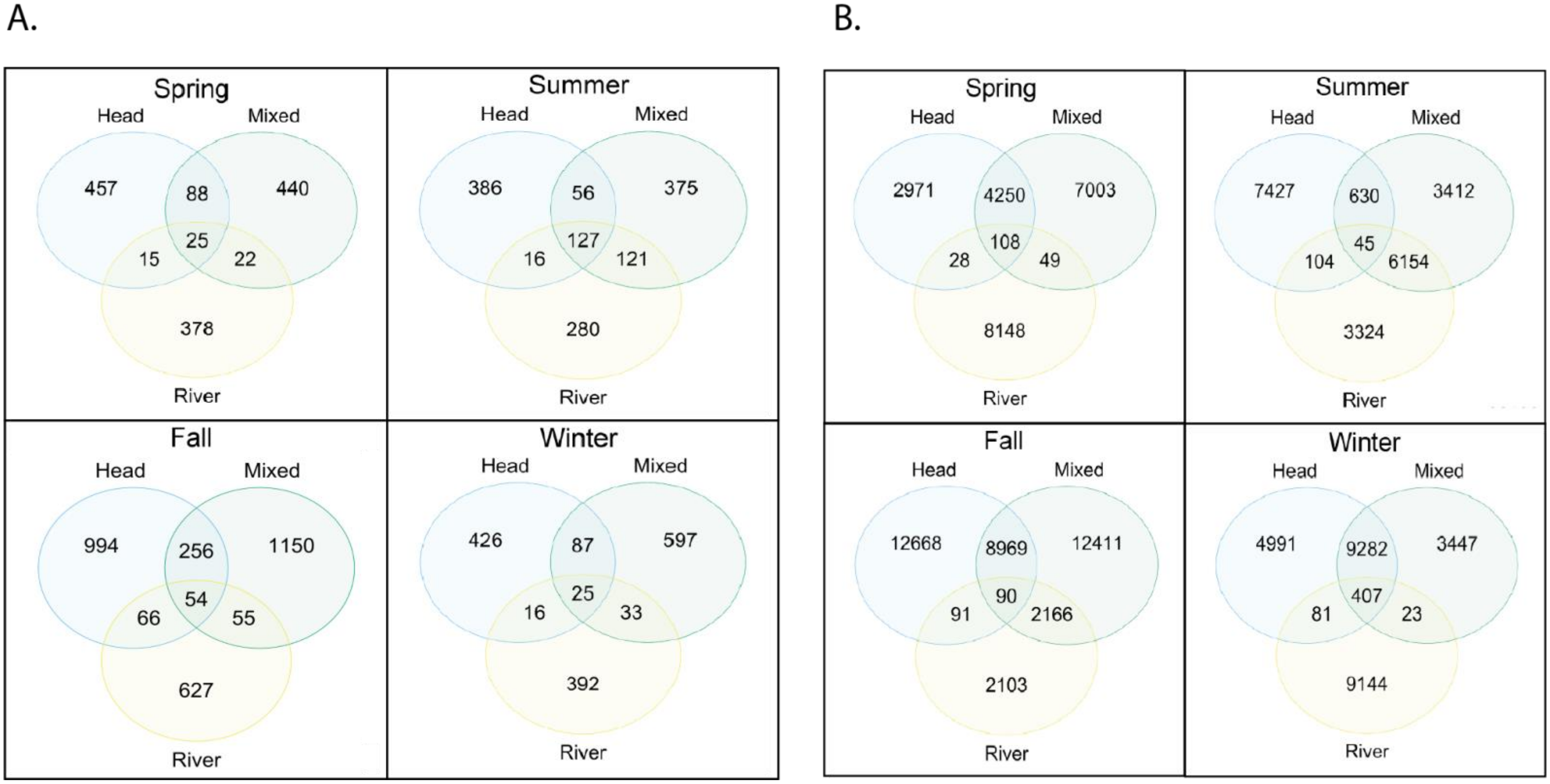

Supplemental Figure 1. Supplementary Figure 1. (A) Venn diagram showing the distribution of ASVs amongst sites by collection date. (B) Venn diagram showing the distribution of viral contigs among each site by collection date.

Supplemental Figure 2

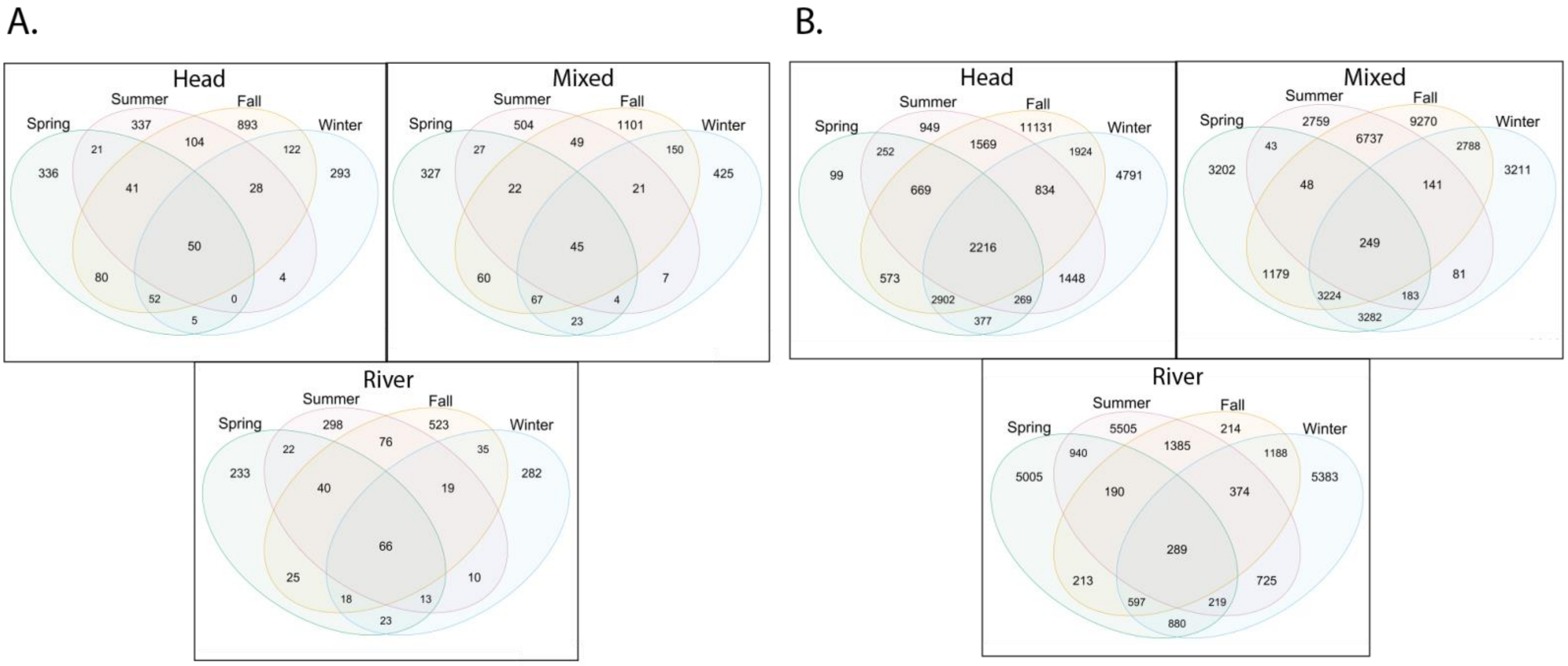

Supplemental Figure 2. (A) Venn diagram showing the distribution of ASVs amongst seasons by collection site. (B) Venn diagram showing the distribution of viral contigs amongst seasons by collection site.

Supplemental Figure 3

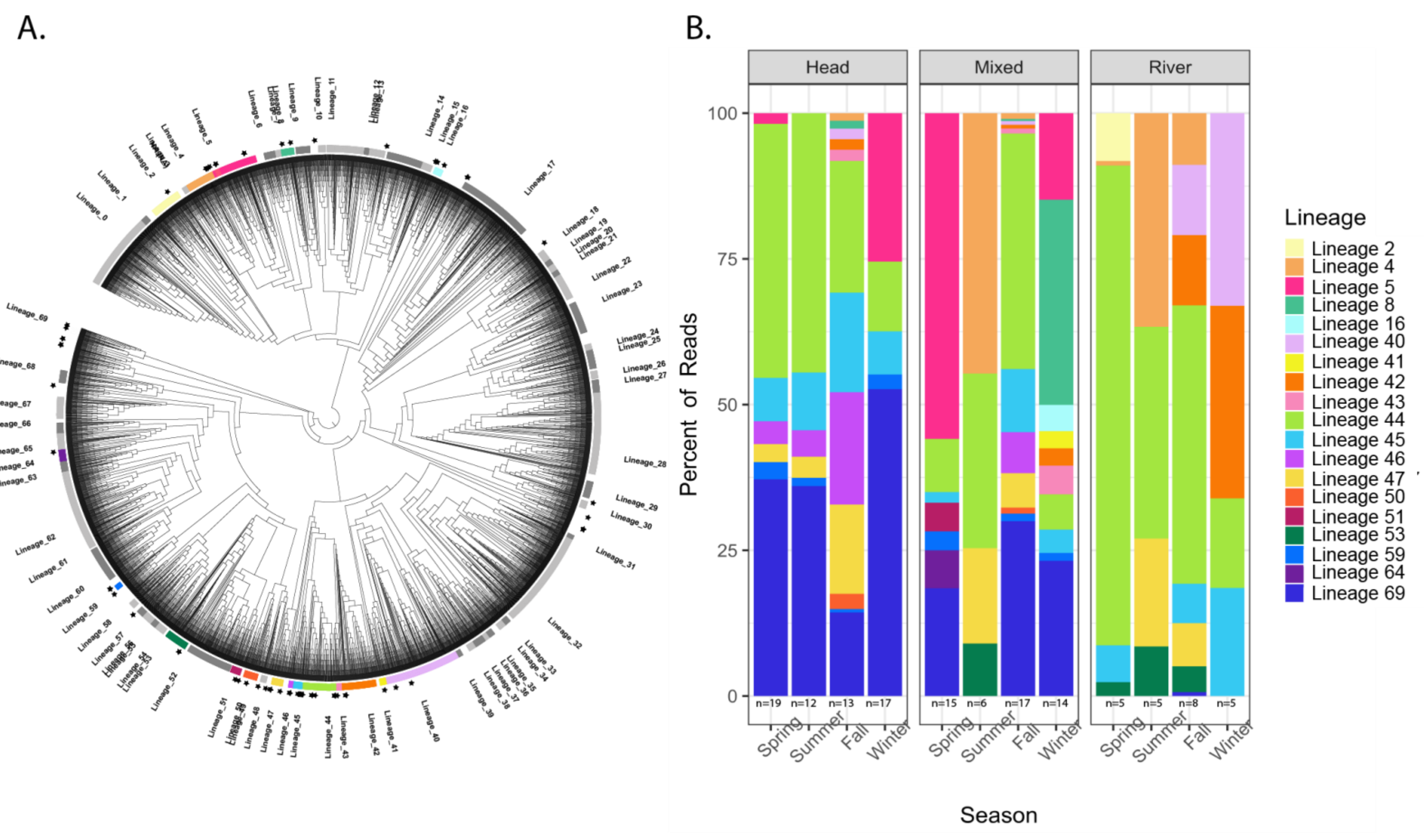

Supplemental Figure 3. (A) Neighbor-joining phylogenetic tree based on Dice distances of prokaryotic viral sequences, depicting lineages established through the GL-UVAB system. Asterisks denote lineages of dsDNA phage analyzed in this study. (B) Stacked bar plot divided by site showing the percent of reads mapping to members of each lineage in each sampling date, n equals the number of complete dsDNA phage genomes in each sample.

Supplemental Figure 4

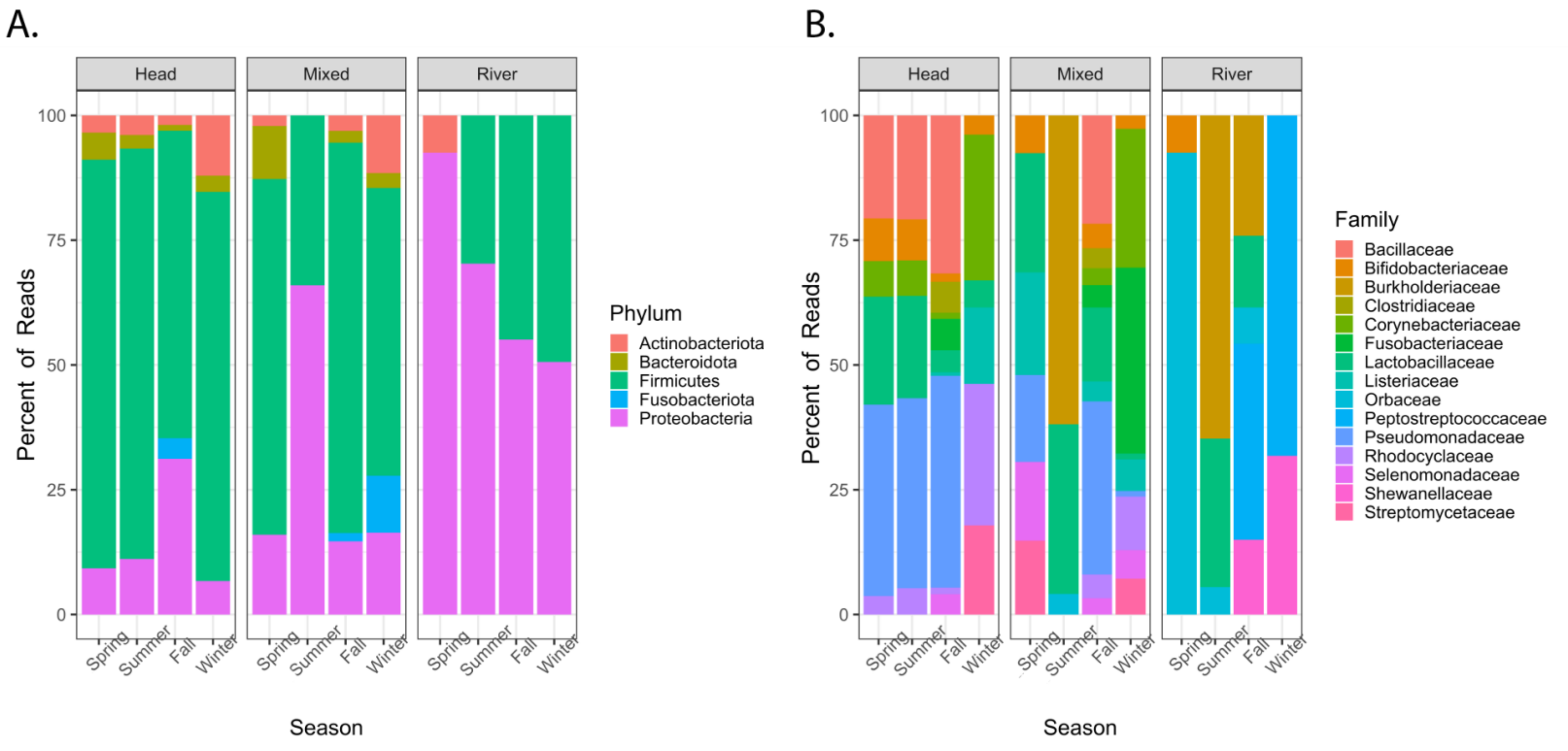

Supplemental Figure 4. (A) Stacked bar plot divided by site showing the relative abundance of predicted hosts by phyla for complete dsDNA phage in each sampling date. (B) Stacked bar plot divided by site showing the relative abundance of predicted hosts by family for complete dsDNA phage in each sampling date.

Supplemental Figure 5

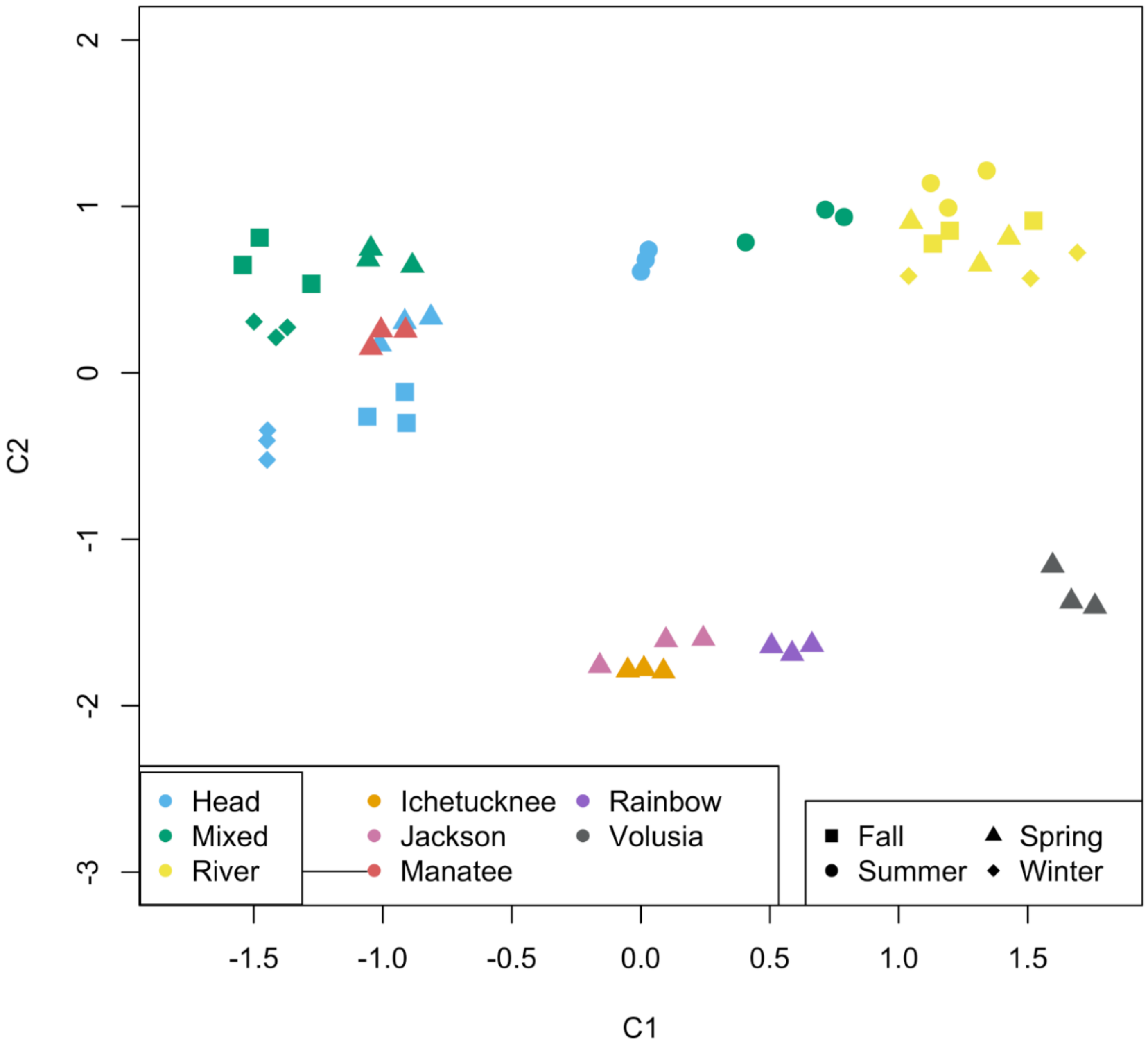

Supplemental Figure 5. NMDS plot showing the similarity of 16S rRNA gene community structure of the spring heads of five first-magnitude springs (1) along with the samples from this study (from various sites and times at Manatee springs) based on a Bray-Curtis dissimilarity matrix of the relative abundance of the ASVs. Season is denoted by shape and color denotes site.

Supplemental Figure 6

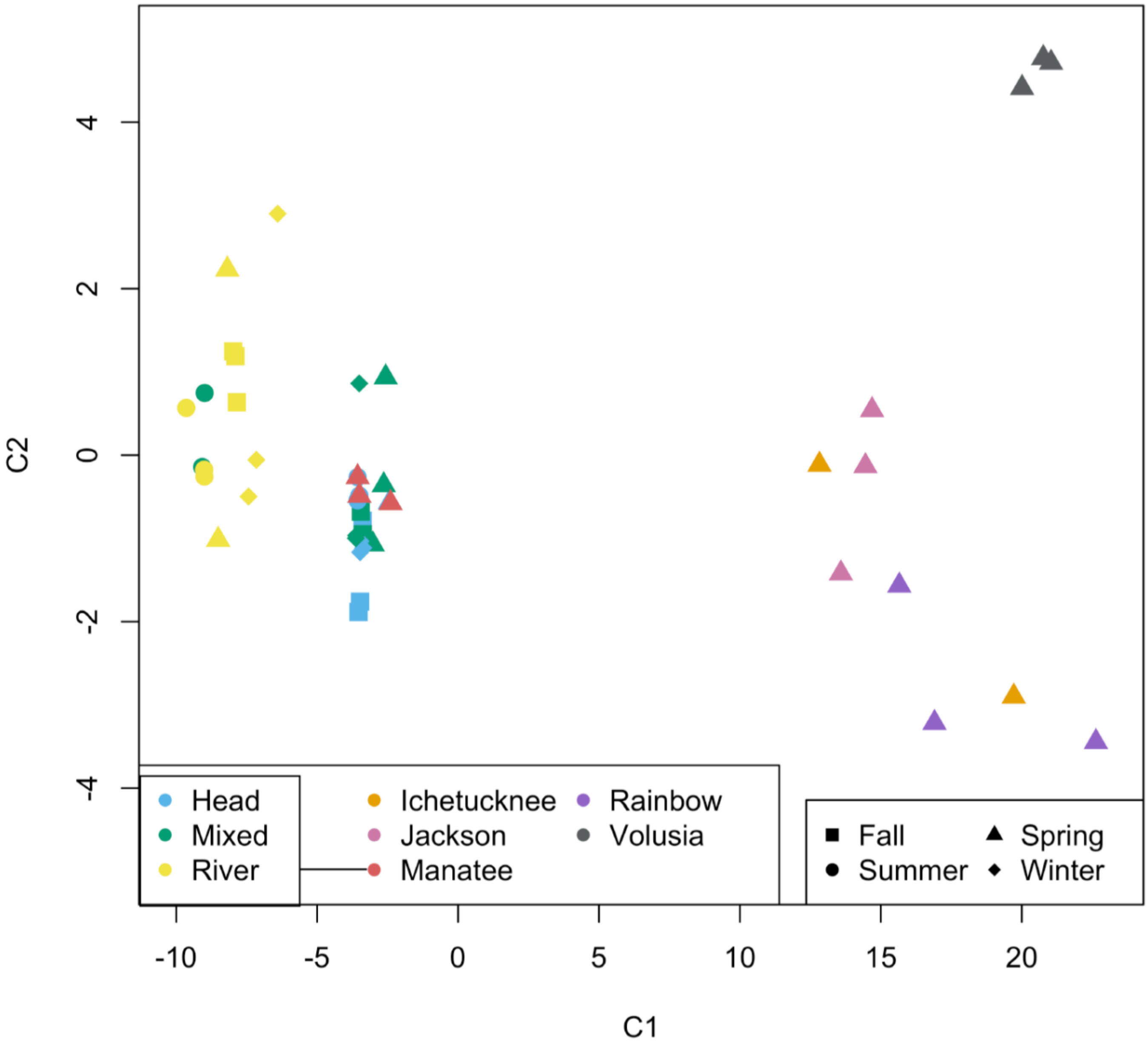

Supplemental Figure 6. NMDS plot showing the similarity of viral community structure of the spring heads of five first magnitude springs (1) along with the samples from this study (from various sites and times at Manatee springs) based on a Bray-Curtis dissimilarity matrix of the relative abundance of viral contigs measured by read coverage normalized by contig length and library size. Season is denoted by shape and color denotes site.

Supplemental Table 2

Name	Accession Number	Type	Lineage	Genome Length (nt)
F_M_2d_ct23	MW202710	Myoviridae	Lineage_44	33873
S_R_2d_ct37962	MW202566	Myoviridae	NA	39561
S_R_2d_ct37972	MW202573	Myoviridae	Lineage_45	35248
SP_H_2d_ct9712	MW202867	Myoviridae	Lineage_5	35140
SP_M_2d_ct3925	MW202426	Myoviridae	NA	50064
SP_M_2d_ct3933	MW202455	Myoviridae	Lineage_64	44651
SP_M_2d_ct3936	MW202897	Myoviridae	Lineage_5	40981
SP_M_2d_ct3945	MW202475	Myoviridae	Lineage_5	40616
SP_R_2d_ct21027	MW202675	Myoviridae	Lineage_4	61820
SP_R_2d_ct21037	MW202711	Myoviridae	Lineage_44	40267
W_H_2d_ct29272	MW202748	Myoviridae	Lineage_5	43596
W_M_2d_ct38315	MW202796	Myoviridae	Lineage_4	44394
W_M_2d_ct38321	MW202496	Myoviridae	Lineage_44	39542
W_R_2d_ct12328	MW202879	Myoviridae	Lineage_44	37068
W_R_2d_ct496	MW202520	Myoviridae	Lineage_45	34188
F_H_2d_ct9	MW202826	Podoviridae	NA	42372
F_M_2d_ct11	MW202572	Podoviridae	Lineage_42	41791
F_M_2d_ct18213	MW202738	Podoviridae	Lineage_47	47384
F_M_2d_ct18222	MW202478	Podoviridae	Lineage_47	44340
F_M_2d_ct18223	MW202482	Podoviridae	NA	43839
F_M_2d_ct18225	MW202744	Podoviridae	Lineage_46	43436
F_M_2d_ct18228	MW202785	Podoviridae	Lineage_4	40616
F_M_2d_ct18229	MW202691	Podoviridae	Lineage_45	41097
F_M_2d_ct18234	MW202801	Podoviridae	NA	40755
F_M_2d_ct45200	MW202733	Podoviridae	Lineage_40	45983
F_M_2d_ct45202	MW202473	Podoviridae	Lineage_47	45228
F_M_2d_ct45215	MW202485	Podoviridae	NA	39021
F_M_2d_ct8	MW202795	Podoviridae	NA	43110
S_H_2d_ct13250	MW202615	Podoviridae	Lineage_69	65827
S_H_2d_ct13253	MW202665	Podoviridae	Lineage_46	41171
S_M_2d_ct12	MW202713	Podoviridae	Lineage_47	43243
S_M_2d_ct16	MW202627	Podoviridae	NA	42295
S_M_2d_ct22	MW202640	Podoviridae	Lineage_53	40083
SP_H_2d_ct26349	MW202898	Podoviridae	Lineage_42	39288
SP_H_2d_ct9704	MW202512	Podoviridae	Lineage_44	44957
SP_H_2d_ct9705	MW202481	Podoviridae	Lineage_44	41808
SP_M_2d_ct3928	MW202647	Podoviridae	NA	48165
SP_M_2d_ct3938	MW202803	Podoviridae	NA	42637
SP_R_2d_ct15	MW202674	Podoviridae	Lineage_45	37201

SP_R_2d_ct21026	MW202828	Podoviridae	Lineage_2	61444
SP_R_2d_ct21034	MW202484	Podoviridae	NA	42333
SP_R_2d_ct9	MW202722	Podoviridae	Lineage_53	40462
W_H_2d_ct29270	MW202554	Podoviridae	NA	44953
W_H_2d_ct29277	MW202483	Podoviridae	Lineage_45	39395
W_M_2d_ct10	MW202477	Podoviridae	NA	42105
W_M_2d_ct17528	MW202697	Podoviridae	Lineage_41	45674
W_M_2d_ct17529	MW202692	Podoviridae	Lineage_16	45513
W_M_2d_ct17533	MW202510	Podoviridae	Lineage_43	42304
W_M_2d_ct8	MW202524	Podoviridae	NA	43134
W_R_2d_ct12313	MW202701	Podoviridae	Lineage_40	55377
F_M_2d_ct18215	MW202777	Siphoviridae	NA	45844
F_M_2d_ct18216	MW202679	Siphoviridae	Lineage_8	45689
F_R_2d_ct18057	MW202789	Siphoviridae	NA	44579
S_H_2d_ct22459	MW202864	Siphoviridae	NA	56879
SP_H_2d_ct26347	MW202604	Siphoviridae	NA	46886
SP_M_2d_ct3937	MW202458	Siphoviridae	NA	42746
SP_R_2d_ct21025	MW202476	Siphoviridae	NA	77627
SP_R_2d_ct21031	MW202423	Siphoviridae	NA	37393
W_H_2d_ct29273	MW202497	Siphoviridae	NA	43554
W_M_2d_ct17524	MW202621	Siphoviridae	Lineage_8	56319
W_M_2d_ct17537	MW202577	Siphoviridae	NA	40259
W_M_2d_ct38312	MW202545	Siphoviridae	Lineage_4	58020
F_H_2d_ct19	MW202655	Misc. Phage	Lineage_45	39148
F_M_2d_ct18231	MW202444	Misc. Phage	Lineage_50	41947
F_M_2d_ct18233	MW202489	Misc. Phage	NA	40925
F_R_2d_ct32458	MW202825	Misc. Phage	NA	34781
SP_M_2d_ct3931	MW202658	Misc. Phage	Lineage_59	45239
SP_R_2d_ct20	MW202753	Misc. Phage	NA	36230
SP_R_2d_ct23	MW202544	Misc. Phage	Lineage_44	35375
F_H_2d_ct10424	MW202784	CRESS	NA	1982
F_H_2d_ct1156	MW202553	CRESS	NA	4595
F_H_2d_ct1197	MW202494	CRESS	NA	3881
F_H_2d_ct1557	MW202625	CRESS	NA	3161
F_H_2d_ct1638	MW202543	CRESS	NA	3297
F_H_2d_ct2415	MW202498	CRESS	NA	3396
F_H_2d_ct2579	MW202488	CRESS	NA	3288
F_H_2d_ct3524	MW202716	CRESS	NA	2887
F_H_2d_ct3707	MW202765	CRESS	NA	2873
F_H_2d_ct4421	MW202734	CRESS	NA	2261
F_H_2d_ct4474	MW202635	CRESS	NA	2652
F_H_2d_ct4637	MW202810	CRESS	NA	2589

F_H_2d_ct4802	MW202563	CRESS	NA	2247
F_H_2d_ct4819	MW202448	CRESS	NA	2602
F_H_2d_ct7404	MW202848	CRESS	NA	2147
F_H_2d_ct850	MW202740	CRESS	NA	5300
F_H_2d_ct8756	MW202433	CRESS	NA	2056
F_H_2d_ct935	MW202490	CRESS	NA	4270
F_H_2d_ct9976	MW202624	CRESS	NA	1965
F_M_2d_ct20239	MW202814	CRESS	NA	3918
F_M_2d_ct20671	MW202775	CRESS	NA	3974
F_M_2d_ct21671	MW202761	CRESS	NA	3367
F_M_2d_ct2188	MW202813	CRESS	NA	3301
F_M_2d_ct2214	MW202845	CRESS	NA	3318
F_M_2d_ct22352	MW202531	CRESS	NA	3084
F_M_2d_ct22959	MW202682	CRESS	NA	2942
F_M_2d_ct23219	MW202431	CRESS	NA	2859
F_M_2d_ct23274	MW202709	CRESS	NA	2859
F_M_2d_ct23461	MW202619	CRESS	NA	2846
F_M_2d_ct23588	MW202798	CRESS	NA	2757
F_M_2d_ct23836	MW202578	CRESS	NA	2711
F_M_2d_ct27852	MW202506	CRESS	NA	2235
F_M_2d_ct45873	MW202729	CRESS	NA	6498
F_M_2d_ct47006	MW202820	CRESS	NA	4066
F_M_2d_ct4704	MW202586	CRESS	NA	2427
F_M_2d_ct47947	MW202736	CRESS	NA	3327
F_M_2d_ct48164	MW202840	CRESS	NA	3221
F_M_2d_ct50483	MW202417	CRESS	NA	2632
F_M_2d_ct52885	MW202858	CRESS	NA	2264
F_M_2d_ct853	MW202599	CRESS	NA	3490
F_R_2d_ct18626	MW202616	CRESS	NA	5050
F_R_2d_ct20117	MW202606	CRESS	NA	3006
F_R_2d_ct21179	MW202519	CRESS	NA	2474
F_R_2d_ct23455	MW202523	CRESS	NA	2096
F_R_2d_ct24983	MW202880	CRESS	NA	1864
F_R_2d_ct33700	MW202792	CRESS	NA	2691
F_R_2d_ct3422	MW202730	CRESS	NA	2507
F_R_2d_ct6876	MW202644	CRESS	NA	2007
S_H_2d_ct14866	MW202706	CRESS	NA	2907
S_H_2d_ct17075	MW202696	CRESS	NA	1769
S_H_2d_ct17906	MW202704	CRESS	NA	1919
S_H_2d_ct2020	MW202708	CRESS	NA	3257
S_H_2d_ct23192	MW202756	CRESS	NA	4794
S_H_2d_ct25745	MW202474	CRESS	NA	2508

S_H_2d_ct26313	MW202689	CRESS	NA	2261
S_H_2d_ct26476	MW202441	CRESS	NA	2103
S_H_2d_ct26956	MW202595	CRESS	NA	2097
S_H_2d_ct3192	MW202422	CRESS	NA	2552
S_H_2d_ct3272	MW202681	CRESS	NA	2521
S_H_2d_ct4352	MW202745	CRESS	NA	2262
S_H_2d_ct5117	MW202875	CRESS	NA	2078
S_H_2d_ct5756	MW202836	CRESS	NA	2036
S_M_2d_ct13218	MW202700	CRESS	NA	1770
S_M_2d_ct2824	MW202593	CRESS	NA	3339
S_M_2d_ct32596	MW202905	CRESS	NA	2526
S_M_2d_ct4531	MW202467	CRESS	NA	2599
S_M_2d_ct47561	MW202660	CRESS	NA	2410
S_M_2d_ct5561	MW202514	CRESS	NA	2442
S_M_2d_ct5783	MW202677	CRESS	NA	2401
S_M_2d_ct69177	MW202794	CRESS	NA	4888
S_M_2d_ct69406	MW202885	CRESS	NA	3522
S_M_2d_ct69971	MW202804	CRESS	NA	3031
S_M_2d_ct70651	MW202469	CRESS	NA	3294
S_M_2d_ct70726	MW202667	CRESS	NA	3220
S_M_2d_ct71506	MW202610	CRESS	NA	2868
S_M_2d_ct74860	MW202505	CRESS	NA	2131
S_M_2d_ct74893	MW202800	CRESS	NA	2050
S_M_2d_ct76125	MW202817	CRESS	NA	1980
S_R_2d_ct30960	MW202555	CRESS	NA	1857
S_R_2d_ct38881	MW202415	CRESS	NA	4906
S_R_2d_ct40851	MW202643	CRESS	NA	3024
S_R_2d_ct41181	MW202602	CRESS	NA	3005
S_R_2d_ct44216	MW202788	CRESS	NA	2339
S_R_2d_ct46756	MW202757	CRESS	NA	2091
S_R_2d_ct5449	MW202603	CRESS	NA	2628
S_R_2d_ct6905	MW202589	CRESS	NA	2417
S_R_2d_ct7793	MW202896	CRESS	NA	2242
SP_H_2d_ct10522	MW202659	CRESS	NA	1831
SP_H_2d_ct12636	MW202764	CRESS	NA	2656
SP_H_2d_ct12727	MW202668	CRESS	NA	2660
SP_H_2d_ct14226	MW202646	CRESS	NA	2010
SP_H_2d_ct14471	MW202437	CRESS	NA	2187
SP_H_2d_ct2237	MW202904	CRESS	NA	2383
SP_H_2d_ct26456	MW202807	CRESS	NA	3560
SP_H_2d_ct603	MW202860	CRESS	NA	3800
SP_M_2d_ct10455	MW202472	CRESS	NA	2507

SP_M_2d_ct11092	MW202874	CRESS	NA	2404
SP_M_2d_ct11979	MW202607	CRESS	NA	2332
SP_M_2d_ct12362	MW202839	CRESS	NA	2237
SP_M_2d_ct1312	MW202592	CRESS	NA	2056
SP_M_2d_ct14136	MW202902	CRESS	NA	2173
SP_M_2d_ct15131	MW202673	CRESS	NA	2116
SP_M_2d_ct16084	MW202574	CRESS	NA	1962
SP_M_2d_ct3174	MW202565	CRESS	NA	4830
SP_M_2d_ct3400	MW202620	CRESS	NA	2218
SP_M_2d_ct3436	MW202833	CRESS	NA	1947
SP_M_2d_ct423	MW202513	CRESS	NA	3498
SP_M_2d_ct4250	MW202780	CRESS	NA	6313
SP_M_2d_ct501	MW202834	CRESS	NA	3272
SP_M_2d_ct5173	MW202525	CRESS	NA	3704
SP_M_2d_ct5991	MW202547	CRESS	NA	4230
SP_M_2d_ct6134	MW202503	CRESS	NA	3900
SP_M_2d_ct7136	MW202452	CRESS	NA	3387
SP_M_2d_ct7222	MW202802	CRESS	NA	3333
SP_M_2d_ct7311	MW202683	CRESS	NA	3386
SP_M_2d_ct7540	MW202705	CRESS	NA	3167
SP_M_2d_ct8558	MW202429	CRESS	NA	2912
SP_M_2d_ct902	MW202533	CRESS	NA	2487
SP_M_2d_ct9275	MW202450	CRESS	NA	2803
SP_R_2d_ct1648	MW202522	CRESS	NA	4037
SP_R_2d_ct21291	MW202570	CRESS	NA	9643
SP_R_2d_ct21566	MW202662	CRESS	NA	3954
SP_R_2d_ct21772	MW202516	CRESS	NA	5428
SP_R_2d_ct22168	MW202571	CRESS	NA	4455
SP_R_2d_ct22938	MW202629	CRESS	NA	3610
SP_R_2d_ct23714	MW202436	CRESS	NA	3091
SP_R_2d_ct23974	MW202844	CRESS	NA	2945
SP_R_2d_ct24103	MW202831	CRESS	NA	2930
SP_R_2d_ct24291	MW202695	CRESS	NA	2877
SP_R_2d_ct24765	MW202551	CRESS	NA	2730
SP_R_2d_ct24831	MW202684	CRESS	NA	2714
SP_R_2d_ct24838	MW202815	CRESS	NA	2707
SP_R_2d_ct24912	MW202793	CRESS	NA	2620
SP_R_2d_ct24914	MW202799	CRESS	NA	2688
SP_R_2d_ct25245	MW202846	CRESS	NA	2566
SP_R_2d_ct25697	MW202811	CRESS	NA	2460
SP_R_2d_ct26072	MW202731	CRESS	NA	2418
SP_R_2d_ct26170	MW202411	CRESS	NA	2431

SP_R_2d_ct27695	MW202767	CRESS	NA	1893
SP_R_2d_ct2874	MW202685	CRESS	NA	3218
SP_R_2d_ct29472	MW202421	CRESS	NA	2010
SP_R_2d_ct30267	MW202899	CRESS	NA	1868
SP_R_2d_ct3338	MW202539	CRESS	NA	3033
SP_R_2d_ct39981	MW202428	CRESS	NA	5521
SP_R_2d_ct5252	MW202617	CRESS	NA	2534
SP_R_2d_ct824	MW202854	CRESS	NA	5507
W_H_2d_ct1516	MW202717	CRESS	NA	3135
W_H_2d_ct21672	MW202612	CRESS	NA	2192
W_H_2d_ct29959	MW202537	CRESS	NA	4719
W_H_2d_ct30765	MW202778	CRESS	NA	3539
W_H_2d_ct32272	MW202776	CRESS	NA	2654
W_H_2d_ct32430	MW202650	CRESS	NA	2664
W_H_2d_ct32889	MW202664	CRESS	NA	2438
W_M_2d_ct17689	MW202851	CRESS	NA	8285
W_M_2d_ct18653	MW202873	CRESS	NA	3663
W_M_2d_ct19685	MW202850	CRESS	NA	3390
W_M_2d_ct19820	MW202424	CRESS	NA	3359
W_M_2d_ct19879	MW202690	CRESS	NA	3279
W_M_2d_ct20571	MW202656	CRESS	NA	3005
W_M_2d_ct22371	MW202528	CRESS	NA	2475
W_M_2d_ct39535	MW202442	CRESS	NA	5189
W_M_2d_ct41659	MW202529	CRESS	NA	2382
W_M_2d_ct41730	MW202686	CRESS	NA	3154
W_M_2d_ct44746	MW202868	CRESS	NA	2431
W_M_2d_ct920	MW202579	CRESS	NA	4447
W_R_2d_ct1107	MW202440	CRESS	NA	3749
W_R_2d_ct12943	MW202822	CRESS	NA	6807
W_R_2d_ct13163	MW202829	CRESS	NA	5562
W_R_2d_ct13272	MW202878	CRESS	NA	3129
W_R_2d_ct13665	MW202890	CRESS	NA	4953
W_R_2d_ct14022	MW202832	CRESS	NA	4434
W_R_2d_ct14028	MW202759	CRESS	NA	3099
W_R_2d_ct14250	MW202416	CRESS	NA	4223
W_R_2d_ct14723	MW202515	CRESS	NA	3838
W_R_2d_ct14776	MW202638	CRESS	NA	3552
W_R_2d_ct14820	MW202707	CRESS	NA	3183
W_R_2d_ct15242	MW202908	CRESS	NA	3525
W_R_2d_ct15272	MW202443	CRESS	NA	3514
W_R_2d_ct15564	MW202720	CRESS	NA	3408
W_R_2d_ct16855	MW202669	CRESS	NA	2935

W_R_2d_ct17236	MW202714	CRESS	NA	2106
W_R_2d_ct17598	MW202688	CRESS	NA	2771
W_R_2d_ct17967	MW202582	CRESS	NA	2649
W_R_2d_ct18809	MW202460	CRESS	NA	2346
W_R_2d_ct19292	MW202865	CRESS	NA	2523
W_R_2d_ct20201	MW202517	CRESS	NA	2332
W_R_2d_ct20423	MW202564	CRESS	NA	2257
F_M_2d_ct4579	MW202694	Inoviridae	NA	2423
S_M_2d_ct69047	MW202766	Inoviridae	NA	5134
S_R_2d_ct39797	MW202783	Inoviridae	NA	3601
W_R_2d_ct12890	MW202693	Inoviridae	NA	7004
W_R_2d_ct13136	MW202882	Inoviridae	NA	6094
F_H_2d_ct1028	MW202464	Microviridae	NA	4848
F_H_2d_ct867	MW202549	Microviridae	NA	5196
F_M_2d_ct19465	MW202895	Microviridae	NA	5534
F_M_2d_ct19794	MW202466	Microviridae	NA	4885
F_M_2d_ct19836	MW202596	Microviridae	NA	4803
F_M_2d_ct451	MW202561	Microviridae	NA	4934
F_M_2d_ct45955	MW202703	Microviridae	NA	6085
F_M_2d_ct46119	MW202609	Microviridae	NA	4771
F_M_2d_ct46239	MW202575	Microviridae	NA	5231
F_M_2d_ct46267	MW202535	Microviridae	NA	5160
F_M_2d_ct527	MW202568	Microviridae	NA	6570
F_M_2d_ct661	MW202581	Microviridae	NA	5618
F_M_2d_ct892	MW202445	Microviridae	NA	4997
F_R_2d_ct18579	MW202768	Microviridae	NA	5285
S_H_2d_ct23142	MW202414	Microviridae	NA	4956
S_H_2d_ct23212	MW202843	Microviridae	NA	4754
S_M_2d_ct1134	MW202590	Microviridae	NA	5065
S_M_2d_ct69068	MW202587	Microviridae	NA	5111
S_R_2d_ct38678	MW202883	Microviridae	NA	5387
S_R_2d_ct38908	MW202671	Microviridae	NA	4856
SP_H_2d_ct10266	MW202597	Microviridae	NA	5169
SP_H_2d_ct10373	MW202680	Microviridae	NA	4754
SP_M_2d_ct5140	MW202889	Microviridae	NA	5462
SP_M_2d_ct5198	MW202760	Microviridae	NA	5280
SP_M_2d_ct5236	MW202847	Microviridae	NA	5219
SP_M_2d_ct5299	MW202558	Microviridae	NA	5081
SP_R_2d_ct21882	MW202732	Microviridae	NA	5114
SP_R_2d_ct21915	MW202471	Microviridae	NA	5016
W_H_2d_ct29835	MW202567	Microviridae	NA	5353
W_M_2d_ct17793	MW202893	Microviridae	NA	5362

W_M_2d_ct18492	MW202819	Microviridae	NA	4810
W_M_2d_ct39392	MW202491	Microviridae	NA	5518
W_R_2d_ct12872	MW202618	Microviridae	NA	7183
W_R_2d_ct13541	MW202853	Microviridae	NA	5111
W_R_2d_ct799	MW202869	Microviridae	NA	4811

Supplemental Table 2. List of complete viral genomes, Genbank accession numbers, taxonomic assignments, lineage assignments, and genome length. NA means no lineage assigned.

References

1. **Malki K, Rosario K, Sawaya NA, Székely AJ, Tisza MJ, Breitbart M.** 2020. Prokaryotic and viral community composition of freshwater springs in Florida, USA. mBio **11:**e00436–20.
